# Recent Advances in Graphene-Based Humidity Sensors

**DOI:** 10.3390/nano9030422

**Published:** 2019-03-12

**Authors:** Chao Lv, Cun Hu, Junhong Luo, Shuai Liu, Yan Qiao, Zhi Zhang, Jiangfeng Song, Yan Shi, Jinguang Cai, Akira Watanabe

**Affiliations:** 1 Institute of Materials, China Academy of Engineering Physics, Jiangyou 621908, China; lvchao219@foxmail.com (C.L.); hucun402@163.com (C.H.); luojunhong@caep.cn (J.L.); zhangzhi@caep.cn (Z.Z.); songjiangfeng@caep.cn (J.S.); shiyan@caep.cn (Y.S.); 2 Institute of Multidisciplinary Research for Advanced Materials, Tohoku University, 2-1-1 Katahira, Aoba-ku, Sendai 980-8577, Japan; 3 School of Chemistry and Chemical Engineering, Southwest Petroleum University, Chengdu 610500, China; shuailiu@swpu.edu.cn; 4 College of Chemistry and Molecular Engineering, Zhengzhou University, Zhengzhou 450001, China; yanqiao@zzu.edu.cn

**Keywords:** humidity sensors, graphene, graphene oxide, reduced graphene oxide, chemical modified graphene, graphene/polymer, graphene quantum dots, graphene/metal oxide, graphene/2D materials

## Abstract

Humidity sensors are a common, but important type of sensors in our daily life and industrial processing. Graphene and graphene-based materials have shown great potential for detecting humidity due to their ultrahigh specific surface areas, extremely high electron mobility at room temperature, and low electrical noise due to the quality of its crystal lattice and its very high electrical conductivity. However, there are still no specific reviews on the progresses of graphene-based humidity sensors. This review focuses on the recent advances in graphene-based humidity sensors, starting from an introduction on the preparation and properties of graphene materials and the sensing mechanisms of seven types of commonly studied graphene-based humidity sensors, and mainly summarizes the recent advances in the preparation and performance of humidity sensors based on pristine graphene, graphene oxide, reduced graphene oxide, graphene quantum dots, and a wide variety of graphene based composite materials, including chemical modification, polymer, metal, metal oxide, and other 2D materials. The remaining challenges along with future trends in high-performance graphene-based humidity sensors are also discussed.

## 1. Introduction

Humidity sensors are a common type of sensors in our daily life, and play a significant role in numerous application fields ranging from humidity control for various kinds of industrial processing, agricultural moisture monitoring, and medical fields to weather forecasting, indoor humidity sensing, and domestic machine controlling [1,2,3,4], and the corresponding research has continued for more than 100 years since the 18th century. Simply, humidity sensors show the humidity by converting the amount of water molecules in the environment into a measurable signal. According to the change of the physical parameters after interacting with water molecules, humidity sensors can be categorized into many types, such as the capacitive type, resistive type, impedance type, optic-fiber type, quartz crystal microbalance (QCM) type, surface acoustic wave (SAW) type, resonance type, and so on [3]. Many materials sensitive to water molecules have been developed as sensing materials in humidity sensors, including ceramics, such as Al_2_O_3_, SiO_2_, and spinel compounds [2]; semiconductors, such as TiO_2_ [5,6], SnO_2_ [7,8,9,10], ZnO [11,12,13,14], In_2_O_3_, Si [15], and perovskite compounds [16,17]; polymers, such as polyelectrolytes [18,19], conducting and semiconducting polymers [20], and hydrophilic polymers [21,22,23,24]; 2D materials, such as MoS_2_ [25,26,27], WS_2_ [28,29,30], and black phosphorus [31,32,33,34]; and carbon materials, such as porous carbon [35], carbon nanotubes [36,37], and graphene [38,39].

Among them, graphene is a unique material, which is an atomically thin, planar membrane of carbon atoms arranged in a honeycomb lattice. The unique atomic arrangement brings unique electronic structures, and extraordinary physical and chemical properties, which promote graphene materials’ applications in many fields, including electronics, optoelectronics, spintronics, catalysts, energy generation and storage, molecular separation, and chemical sensors [38,39,40,41,42,43,44,45,46,47]. Graphene and graphene-based materials have shown great potential for the detection of various kinds of gases due to their ultrahigh specific surface areas, extremely high electron mobility at room temperature, and low electrical noise due to the quality of their crystal lattice and very high electrical conductivity [48]. Although many excellent review articles have summarized the progresses on graphene-based chemical, gas, and tactile sensors [38,39,45,46,48,49,50,51,52,53,54], and several review articles have addressed the mechanisms, materials, and development of humidity sensors [1,2,3,4,55,56], there are still no specific reviews on the progresses of graphene-based humidity sensors. This review focuses on the recent advances in graphene-based humidity sensors, which is divided into three main parts: Preparation and properties of graphene, sensing mechanisms of graphene-based humidity sensors, and advances in the humidity sensors based on graphene and graphene composite materials. The first part will briefly introduce three commonly used methods to prepare pristine graphene and graphene oxide, as well as the properties of different kinds of graphene materials. The second part will explain the sensing mechanisms of seven types of commonly studied humidity sensors, including field-effect transistor (FET)-type, resistive, impedance, capacitive, surface acoustic wave (SAW), quartz crystal microbalance (QCM), and optical fiber humidity sensors, and the progresses of SAW, QCM, and optical fiber humidity sensors based on graphene materials will be comprehensively reviewed in this part. The third part mainly focuses on the progresses of graphene-based humidity sensors operating in electronic modes, such as field-effect transistor (FET)-type, resistive, impedance, and capacitive humidity sensors, which are classified into eight sub-parts according to the properties and composites of graphene materials. Finally, the remaining challenges along with future trends in high-performance and practical graphene-based humidity sensors are also discussed.

## 2. Preparation and Properties of Graphene

### 2.1. Preparation of Graphene

The preparation of graphene materials is critical for practical applications. Various methods have been developed to synthesize graphene materials with different structures since the first preparation was accomplished by mechanical exfoliation of graphite in 2004. In this method, Scotch tapes were generally employed to stick the graphite crystals or graphite flakes, and repeatedly peeled over a long period to obtain the graphene layers. This process can also be conducted with a silicon wafer with an oxidized layer. The products are monolayer or few-layer graphene with perfect atomic arrangement, which can be used to fabricate principle electronic devices, such as gas sensors or electronic biosensors [48,57]. However, this method has an extremely low productivity, thus it is difficult to be applied widely [58].

**Figure 1:**
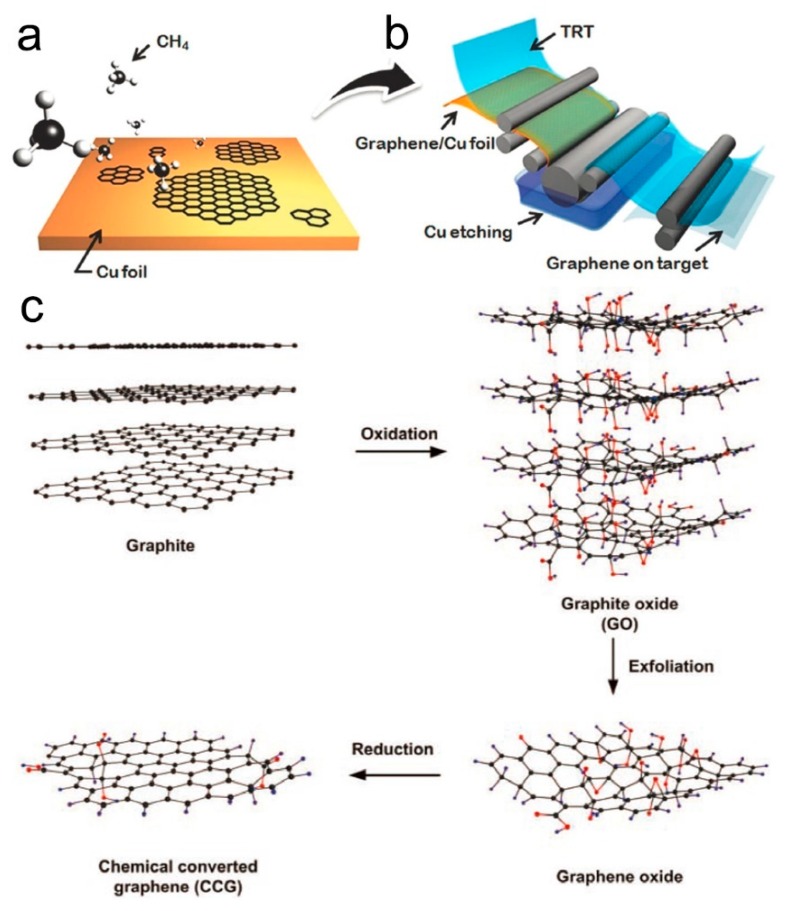
Schematics of the synthesis mechanism of chemical vapor deposition (CVD) graphene on Cu foil (**a**) and the roll-to-roll transfer process of graphene (**b**). Reproduced with permission from [42]. Copyright Wiley-VCH, 2016. Schematic of the preparation of graphene oxide and reduced graphene oxide by the reduction of graphene oxide (**c**). Reproduced with permission from [63]. Copyright Wiley-VCH, 2011.

Fortunately, the chemical vapor deposition (CVD) technique, which was widely used in the preparation of one-dimensional (1D) structures, has been developed and extensively explored to grow high-quality graphene with superior properties [59]. Like the growth process of 1D nanostructures, the growth of graphene includes three main steps, the dissolution of carbon atoms into the metal substrate at higher temperature, the precipitation of carbon from the substrate in the cooling stage caused by the reduced solubility, and the formation of graphene layers on the metal surface [60]. Some metals, such as Ni, Cu, Fe, Co, Ir, Pt, and Ni-Mo alloy, have been developed as a catalyst and substrate to grow graphene layers. The growth of single-layer or multilayer graphene films can be controlled by using metals with different solubility of carbon atoms. For example, multilayer graphene films are usually prepared on Fe, Ni, and Co substrates with relatively high solubility [61], while single-layer graphene films can grow on Pt and Cu substrates due to the relatively low solubility (Figure 1a). To use the graphene layers in electronic devices, the effective transfer technology of the chemical wet-etching method was developed to dissolve the metal substrate and transfer the graphene films, and this process does not produce defects or degrade graphene properties. This process was further developed in a roll-to-roll way for continuous and large-area production (Figure 1b) [62]. Nevertheless, more facile, effective, and low-cost technologies are urgently required for large-scale commercial applications.

Another common strategy of the solution-processed approach, which is also called the Hummers method, has been widely used in the massive and reproducible preparation of graphene materials [40]. This approach involves two main steps, the oxidation and exfoliation of graphite in liquid solution and the formation of graphene by reduction. In the first step, the graphite powder is oxidized by strong acids and oxidants, followed by exfoliation under sonication, producing hydrosoluble graphene oxide (GO) (Figure 1c) [63]. GO with different layers can be obtained by purification and separation by centrifugation. Then, reduced graphene oxide (rGO) can be obtained through chemical reduction, thermal reduction, microwave irradiation, electrochemical reduction, photo reduction, or laser-induced reduction. Compared to the CVD method, which mainly grows single-layer or multilayer graphene films, this method can produce GO or rGO with abundant functional groups in a powder form at a very large scale and with relatively low costs [52,64]. However, this method has some shortages, such as environmental pollution, explosion hazards, and a long period during the oxidation process, and the resultant graphene materials have lots of defects.

### 2.2. Properties of Graphene

The unique atomic thickness and honeycomb lattice structure of sp^2^-bonded carbon atoms, endow graphene with excellent physicochemical, electronic, optical, thermal, and mechanical properties [65]. Graphene can be regarded as a single layer of graphite, but it exhibits a semi-metal feature with a unique zero band gap, different from the metallic behavior of graphite. The charge carriers of graphene have zero rest mass near their Dirac point with a high carrier density of up to 10^13^ cm^−2^, thus graphene exhibits a significant high room-temperature carrier mobility of ~10,000 cm^2^ V^−1^ s^−1^ and a low temperature carrier mobility of 200,000 cm^2^ V^−1^ s^−1^, corresponding to a resistivity of 10^−6^ Ω [65,66]. Due to the perfect atomic arrangement, graphene possesses excellent mechanical properties with fracture strains of up to 25% and a Young’s modulus of ~1.1 TPa [67], even higher than steel’s tensile strength, showing large potential for applications in flexible, stretchable, and wearable electronics. Single-layer graphene has a very high transparency of up to 97.7% with an extremely low theoretical sheet resistance of 30 Ω sq^−1^, showing significant promise in high-performance optic and electronic devices. Additionally, graphene has a very high specific surface area of up to ~2600 m^2^/g [38]. The extremely high electron mobility, the ultrahigh specific surface area to adsorb molecules, and inherently low electronical noise due to the high-quality crystal lattice and very high electrical conductivity make graphene highly sensitive to changes in its chemical environment, thus graphene is recognized as an ideal candidate for the ultrahigh sensitivity detection of gases in various environments [48].

Some of the above properties are only shown in pristine perfect single-layer graphene, which is quite different from graphene materials, such as GO or rGO, prepared by the solution-phase method. In contrast to CVD growth, the solution-phase approach mostly introduces plenty of oxygenic functional groups, such as carboxyl, epoxides, and hydroxyl groups, and defects exist on the graphene basal plane and edges [45]. Thus, as-prepared GO is an electrical insulator with no conductivity, and reduction is necessary to make it conductive in most practical applications. The functional groups, defects, and other unavoidable contamination may degenerate the physical properties. Generally, the conductivity, transparency, and mechanical properties of rGO are several orders lower than those of pristine graphene [40]. Nevertheless, GO and rGO show other advantages. For example, the abundant oxygen containing groups endow GO or rGO with various stimuli-responsive behaviors in aqueous solution. GO and rGO can be further chemically modified to improve their properties for additional functions [45]. The electronic properties of rGO can be modulated by heteroatom doping, and other properties can also be realized by covalently grafting functional groups. In addition, non-covalent interactions between GO or rGO and other components can be employed to prepare graphene-based composite structures for more extensive applications [40]. Furthermore, GO or rGO can be dispersed in solutions and assembled into 1D fibers, 2D films, and 3D porous structures, which diversifies their application areas. Therefore, there is plenty of room to develop and improve the performance of gas sensors, including humidity sensors, based on GO and rGO materials.

## 3. Mechanisms of Graphene-Based Humidity Sensors

Principally, water molecules in the gas environment will adsorb onto the graphene surfaces in a graphene-based humidity sensor, which causes changes of some properties of the graphene materials, corresponding to the humidity change. Various kinds of graphene-based humidity sensors have been developed according to different sensing mechanisms or sensor configurations. This section briefly introduces seven types of sensing mechanisms commonly applied in graphene-based humidity sensors, and the progress of graphene-based humidity sensors working in the last three mechanisms, i.e., SAW, QCM, and optical fiber, is also included.

### 3.1. Field-Effect Transistor (FET) Humidity Sensors

**Figure 2:**
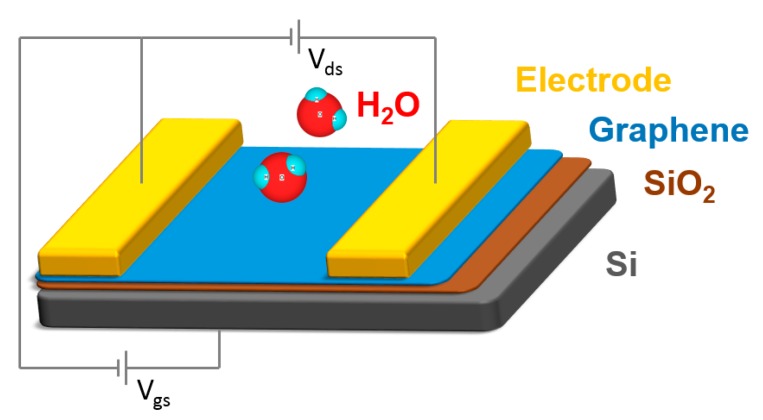
Schematic of a graphene-based field-effect transistor (FET) humidity sensor with source-drain voltage, *V*_ds_, and gate voltage, *V*_gs_, control.

FET configuration has been widely applied in many gas sensing devices due to the simple preparation process, high sensitivity, and portability, as well as the easy miniaturization to the nanoscale [57]. A typical graphene-based FET humidity sensor is comprised of three main parts, graphene as the channel material, two conductive electrodes as the source and drain electrodes, and a gate electrode with a thin dielectric layer (Figure 2). A bias voltage is applied on the gate electrode through the dielectric layer, which can modulate the conductivity of the graphene channel. Humidity sensing is conducted by measuring the current change of the graphene channel before and after exposing it to a humidity environment under a constant applied gate voltage. When adsorbing the water molecules, the electronic structure of the sensing graphene will be changed, leading to a change of the conductivity. In this type of humidity sensor, the sensing material, graphene, can be easily functionalized with different groups or complexed with other components to improve the sensitivity of the humidity sensors. Notably, many gases, including CO, NO, NH_3_, NO_2_, H_2_, SO_2_, H_2_S, and ethanol, can be detected by such a type of gas sensor due to the different interactions between the gas molecules and sensing materials [48,68].

### 3.2. Resistive Humidity Sensors

**Figure 3:**
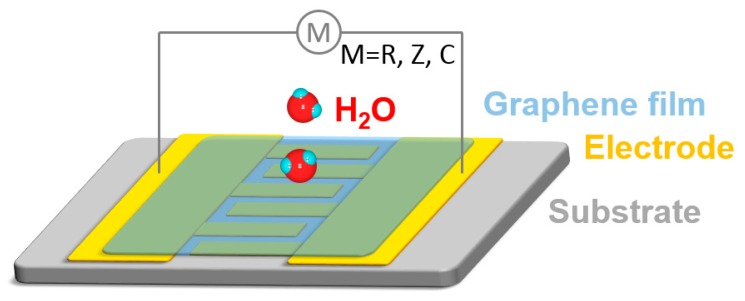
Schematic of a graphene-based humidity sensor in a resistive, impedance, or capacitive working mode depending on the measuring parameter.

Resistive humidity sensors are one of the most widely investigated types of humidity sensing devices because of their many intrinsic advantages, such as the facile fabrication, simplicity of operation, cost effectiveness, reusability, low driven power, and easy miniaturization. The sensing mechanism is based on the change of the electrical resistance of graphene materials caused by the water molecules adsorbed on the surfaces, and the humidity can be effectively determined by measuring the resistance change (Figure 3) [46,69]. Equivalently, the humidity sensor can also detect the current change. The configuration of resistive humidity sensors is relatively simple, which consists of sensing materials located between two conductive electrodes on an inert substrate. The electrodes always employ an interdigitated structure to improve the sensing area and thus the sensitivity. The sensing graphene materials can easily be coated onto the substrate with patterned electrodes through spin-coating, drop-casting, spray-coating, dip-coating, printing, or laser transfer deposition. Generally, the sensitivity of such types of humidity sensors can be improved by increasing the materials, surface areas, as well as the functional groups sensitive to water molecules through constructing porous structures, surface modification, or compositing with other sensitive components. Sensors in this type are also widely employed to detect different kinds of gases, such as CO, H_2_, NO*_x_*, SO*_x_*, O_2_, Cl_2_, and organic vapors, by changing the sensing materials [38,46,48,52,70,71,72].

### 3.3. Impedance Humidity Sensors

Impedance humidity sensors are a type of mixed potential sensors, which work based on impedance change. The humidity sensor is applied with a sinusoidal voltage in the frequency domain, and the impedance is calculated by measuring the current. In such a type of humidity sensor, an impedance spectroscopy is utilized to measure the response of the humidity sensor over frequencies ranging from subhertz to megahertz [73]. There is an advantage in such-type humidity sensors that it has the potential to accurately detect a relatively low humidity, but generally, it requires professional high-performance impedance spectroscopy. The structure of the impedance humidity sensor is similar to the resistive one, in which sensing materials are deposited on two conductive electrodes with gaps on an inert substrate (Figure 3). In impedance humidity sensors, GO and rGO based materials rather than pristine graphene are utilized as sensing materials, because the abundant functional groups on the surfaces as well as porous structures can provide more active sites for water molecules, which induces a simultaneous resistance and capacitance change of the graphene materials, i.e., comprehensive impedance change [74]. Similarly, GO or rGO can be composited with other active components to further improve the sensitivity. Impedance sensors have also been developed for the efficient detection of various gases, including NO_x_, CO, and hydrocarbons, with low concentrations [69].

### 3.4. Capacitive Humidity Sensors

Capacitive humidity sensors utilize the special properties of GO materials, which are insulating, but show proton conductivity when the surfaces adsorb water molecules, with the proton conductivity being related to the water concentration in the environment. This induces the change of the capacitance [75]. The sensor detects the humidity by measuring the capacitance of the device. Therefore, GO can work as a water molecule-sensitive dielectric in a double-layer electrochemical capacitor to detect humidity in the atmosphere. A typical capacitive humidity sensor is generally the in-plane type to expose the maximum active surface area, which consists of two parts, interdigitated conductive electrodes and GO-based sensing materials as the dielectric (Figure 3) [76]. In capacitive humidity sensors, interdigitated conductive electrodes can be metal materials, as well as rGO, which can form all-graphene based humidity sensors. For the dielectric, GO can be composited with other proton conductive components to improve the sensitivity and response [77].

### 3.5. Surface Acoustic Wave (SAW) Humidity Sensors

**Figure 4:**
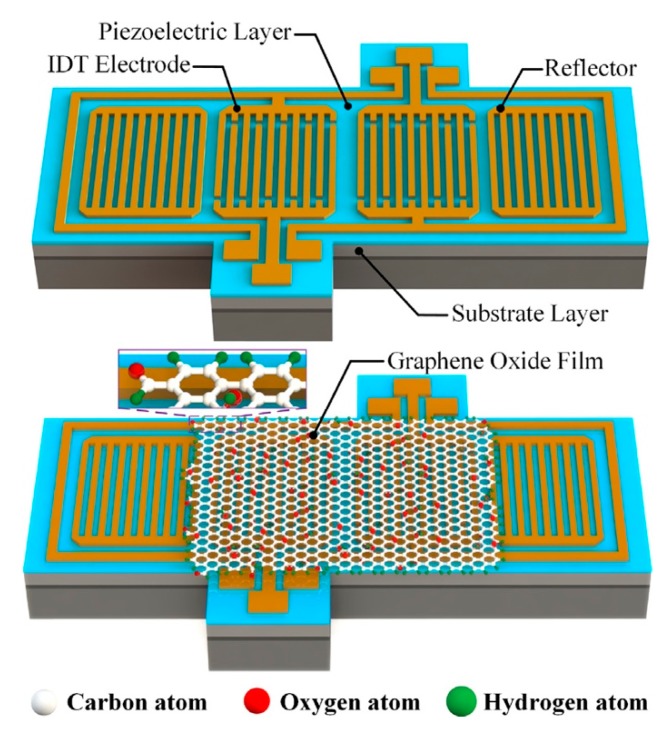
Schematic view of a surface acoustic wave sensor with a clean surface and covered with a graphene oxide (GO) film. Reproduced with permission from [79], Copyright Elsevier B.V., 2017.

Principally, SAW humidity sensors are a type of microelectromechanical system, which detect the humidity change depending on the modulation of surface acoustic waves. An SAW sensor basically comprises a piezoelectric substrate, an input interdigitated transducer (IDT) on one side, a second output IDT on the other side of the substrate, and a delay line in the space between the two IDTs (Figure 4) [78,79]. The input IDT produces a mechanical SAW under the sinusoidal electrical signal using the piezoelectric effect of the piezoelectric substrate, and the SAW propagates across the decay line, where the SAW is influenced by the environment. The SAW is converted into an electric signal by the piezoelectric effect in the output IDT, and any changes made to the mechanical wave are reflected in the output electric signal. The sensing can be accomplished by measuring changes in the amplitude, phase, frequency, or time-delay between the input and output electrical signals. Any changes of the physical or chemical properties of the sensor surface, such as the mass, conductance, or viscoelasticity, affect the acoustic wave velocity or attenuation [80]. Graphene-based SAW humidity sensors generally employ graphene materials as the acoustic layer and utilize the mass change induced SAW change to detect the humidity because hydrophilic GO or GO-based composites enhance the water adsorption capability through the oxygen-rich groups, strengthening the mass loading effect. Based on the above mechanism, SAW sensors towards other gases or targets can also be designed by modulating the properties of the sensing materials and their interaction with sensing targets.

Balashov et al. investigated the sensing performance of SAW humidity sensors by coating a submicro-thick film of GO or a polyvinyl alcohol (PVA) thin film on the decay line, and the GO-based humidity sensor showed a sensitivity of 1.54 kHz/% RH (relative humidity), much higher than the 0.47 kHz/% RH and 0.13 kHz/% RH for the PVA coated and uncoated one, respectively [78]. The SAW humidity sensor prepared on the LiNbO_3_ surface coated with a CVD-growth graphene layer showed a frequency downshift of 1.38 kHz/% RH in the RH range lower than 50% due to the mass loading and a frequency downshift of 2.6 kHz/% RH in the RH range higher than 50% due to the change in the elastic properties of graphene film [81]. The SAW humidity sensor based on GO coated LiNbO_3_ plate showed a super high sensitivity by employing a high-order lamb wave with a large coupling constant, standard LiNbO_3_ plate, and graphene oxide sorbent film, showing a minimal detectable level as low 0.03% RH, and reproducibility of ±2.5% [82]. Xuan et al. demonstrated SAW humidity sensors based on a ZnO piezoelectric thin film on a glass substrate with GO as the sensing layer, which exhibited a high sensitivity from 0.5% RH to the 85% RH range with very fast responses (rise time of 1 s and fall time of 19 s) [83]. Then, they realized a high-performance flexible SAW humidity sensor with the same GO sensing layer on a piezoelectric ZnO thin film deposited on a flexible polyimide substrate [84]. Similarly, Kuznetsova et al. reported an SAW humidity sensor based on a GO film/ZnO film/Si substrate with an enhanced sensitivity of about 91 kHz/% RH and a linear response towards relative humidity in the range of 20–98% [85]. Recently, Xie’s group proposed an SAW humidity sensor based on an AlN/Si (doped) layered structure with a GO sensing layer for a high sensitivity and low temperature coefficient of frequency [79]. The humidity sensor coated with the GO layer exhibited an improved sensitivity of up to 42.08 kHz/% RH in the RH higher than 80% and worked well at both low (<10% RH) and high (>90% RH) humidity. Moreover, it showed low hysteresis, outstanding short-term repeatability, long-term stability, and improved thermal stability.

### 3.6. Quartz Crystal Microbalance (QCM) Humidity Sensors

**Figure 5:**
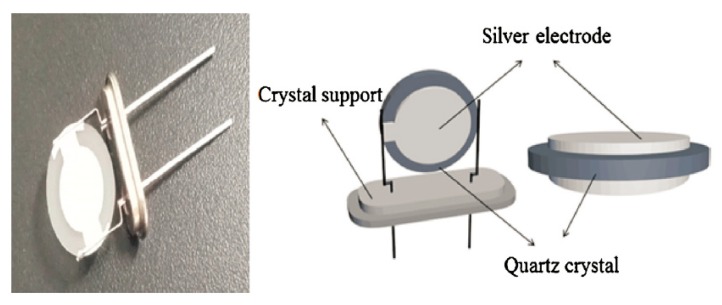
Photograph and structure of the quartz crystal microbalance (QCM) sensor. Reproduced with permission from [86], Copyright Elsevier B.V., 2017.

The QCM technique has been widely used to analyze the mass, membrane structure, molecular interaction, and viscoelasticity changes on the surface of electrodes. QCMs coated with water-sensitive materials can act as sensors for humidity detection, which have the advantages of being ultra-sensitive towards water molecules adsorbed on the crystal surface and a digital frequency output. A QCM humidity sensor can be directly connected to a digital control system to simplify the signal processing circuit. A typical QCM humidity sensor consists of a bare quartz crystal resonator deposited with a humidity sensing material on its electrode, forming a composite resonator (Figure 5) [86]. The composite resonator shows an oscillating frequency shift when the water molecules adsorb or desorb on the sensing material, reflecting the ambient humidity change. There are two types of interaction between water molecules and sensing materials in a QCM humidity sensor, the mass change due to the adsorption and desorption on the surface (mass effect) and the viscoelastic change of the adsorbed film caused by the penetration of water molecules (viscosity effect) [87].

GO is recognized as a promise humidity sensing material because of its abundant hydrophilic oxygen-containing functional groups, such as hydroxyl, carboxyl, and epoxy groups. Yao et al. demonstrated the first GO thin film-coated QCM humidity sensors, which exhibited excellent humidity sensitivity properties with a sensitivity of up to 22.1 Hz/% RH and a linear frequency response in the wide detection range of 6.4–93.5% RH. They found that, at the low RH range (<54.3%), mass changes caused by water molecules’ adsorption/desorption accounted for the frequency response of the sensor, while in the high RH range (>54.3%), both the water adsorption/desorption mass change and expansion stress of the GO thin film induced by the swelling effect caused the frequency change [88]. They also demonstrated that the GO coated QCM humidity sensor showed better stability than the PEG-coated one [89]. The incorporation of other components into the GO film has also been widely investigated to improve the humidity sensing performance by addressing the mass effect or viscosity effect. Other carbon materials, such as multi-walled carbon nanotubes (MWCNTs), nanodiamond, or fullerene, were introduced into the GO film to prevent the viscosity effect, because the intercalation may expand the GO film and improve water molecule diffusion [87,90,91]. Many hygroscopic or hydrophilic components, such as poly (diallyl dimethyl ammonium chloride) [18], poly ethyleneimine and protonated poly ethylenimine [92,93], polyethylene oxide [94], polyaniline [86], SnO_2_ [95], and ZnO [96], have been introduced into GO or rGO films by the direct mixing method or layer-by-layer assembly, which can provide more active sites for water adsorption to improve the humidity sensing performance, including the sensitivity, response/recovery time, hysteresis, repeatability, selectivity, and long-term stability. Additionally, diamine- and β-cyclodextrin-functionalized graphene oxide films exhibited a good response to the low humidity region due to the strong sensitivity of the –CONHC_2_H_4_NH_2_ groups to water molecules [97].

### 3.7. Optical Fiber Humidity Sensors

**Figure 6:**
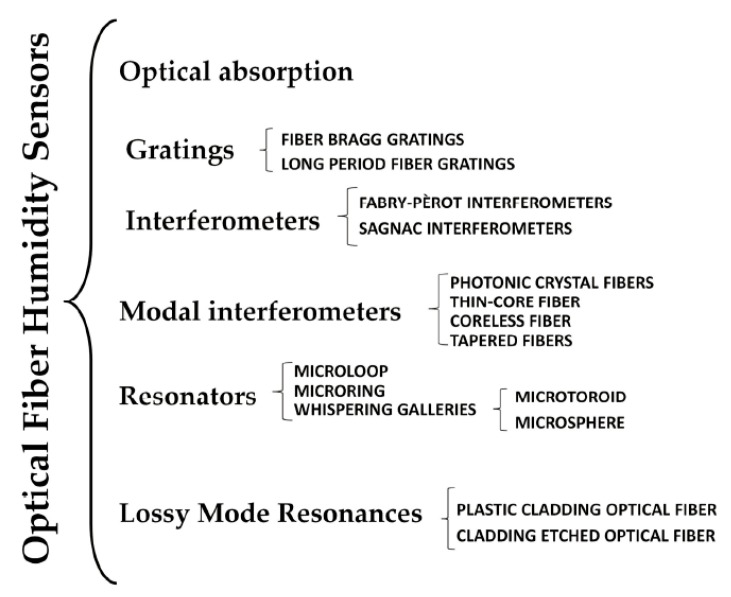
Classification of optical fiber humidity sensors [98].

Optical fiber humidity sensors detect changes of the optical properties inside the fiber caused by water molecules, such as changes of the transmitted optical power, dielectric properties, or the refractive index. Compared to electronic humidity sensors, optical fiber humidity sensors show several advantages, such as the possibility of working in harsh conditions, including flammable environments; higher temperature and pressure ranges; and electromagnetic immunity. However, some facts, including the fabrication repeatability and the high cost of the optical equipment, have prevented the commercial applications of optical fiber humidity sensors. According to the working principle, optical fiber humidity sensors can be classified into several groups, such as humidity sensors based on the optical absorption of materials; humidity sensors based on fiber Bragg gratings and long-period fiber gratings; humidity sensors based on interference (Fabry-Pérot, Sagnac, Mach-Zehnder, Michelson, and modal interferometers); humidity sensors based on micro-tapers, micro-ring, micro-knot resonators, and whispering galleries modes; and humidity sensors based on electromagnetic resonances, specifically lossy mode resonances [98]. The classification is shown in Figure 6. More details can be found in a comprehensive and specialized review on optical fiber humidity sensors summarized by Ascorbe et al. [98], thus this review will not describe the detailed mechanisms.

Graphene materials have the potential to be applied in optical fiber humidity sensors due to the good adsorption towards water molecules, which will induce changes of the optical properties in the optical fiber. For example, Xiao et al. reported an rGO based optical fiber humidity sensor by coating an rGO film on the polished surface of a side-polished fiber, which achieved a power variation of up to 6.9 dB in the high relative humidity range (70–95%) and displayed a linear response with a correlation coefficient of 98.2%, sensitivity of 0.31 dB/% RH, response speed of faster than 0.13% RH/s, and good repeatability in the 75–95% RH [99]. Recently, they demonstrated an improved optical fiber humidity sensor based side-polished single-mode fiber coated with GO film, which exhibited a better performance [100]. The water adsorbed on the GO film caused the dielectric property change, which could influence the TE-mode absorption at 1550 nm of the polymer channel waveguide, constructing the relation between optical absorption and relative humidity [101]. Wang et al. developed humidity sensors based on tilted fiber Bragg grating coated with GO film, which showed a maximum sensitivity of 0.129 dB/% RH with a linear correlation coefficient of 99% under the RH range of 10–80% due to the dependence of the cladding mode resonances on the water absorption and desorption on the GO film [102]. They also investigated optical fiber humidity sensors based on an in-fiber Mach-Zehnder interferometer coated with GO or GO/PVA composite, showing high sensitivity, good stability, and linearity [103,104]. An optical fiber humidity sensor based on a Fabry–Perot resonator was also demonstrated by depositing rGO on the other surface of a hollow core fiber, and the light leakage at the resonant wavelength, which depended on the refractive index of rGO, could be measured to reflect the humidity [105]. Optical fibers coated with graphene materials can be applied not only in humidity sensors, but also in chemical and biological sensors [106].

## 4. Humidity Sensors Based on Graphene Materials

As seen in the previous sections, water molecules in the environment can induce physical and electronic changes in graphene-based sensing materials. The detection of humidity is thus possible by monitoring a variety of properties, such as the resistance, impedance, capacitance, mass, and surface acoustic wave. Previous studies reporting SAW, QCM, and optical fiber sensors were covered in Section 3.5, Section 3.6 and Section 3.7. This section describes recent advances in graphene-based humidity sensors based on electronic properties using measuring techniques, such as FET, resistance, impedance, and capacitance, and it is organized to illustrate the utilization of the different sensor types for the eight sub-classes of graphene materials.

### 4.1. Humidity Sensors Based on Pristine Graphene

**Figure 7:**
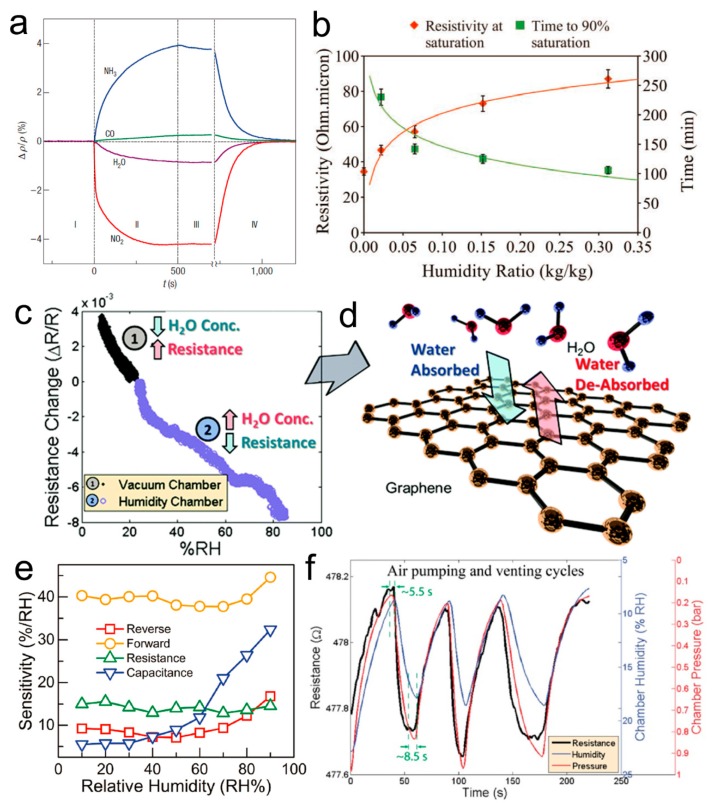
Changes in resistivity caused by graphene’s exposure to various gases diluted in concentration to 1 ppm (**a**). Reproduced with permission from [57], Copyright Nature Publishing Group, 2007. The values of maximum resistivity observed, and the time taken to achieve 90% of the saturation value for different values of absolute humidity (**b**). Reproduced with permission from [107], Copyright Wiley-VCH, 2010. Resistance change in the graphene device versus the relative humidity (%RH) for a device placed in a vacuum chamber (1) and the same device placed in a humidity chamber (2) (**c**) and the interaction of water molecules with the graphene surface (**d**). Reproduced with permission from [108], Copyright Royal Society of Chemistry, 2015. Sensitivity of the humidity sensors under current and capacitance modes (**e**). Reproduced with permission from [111], Copyright Wiley-VCH, 2017. Resistance response of the double-layer graphene device in comparison with the %RH response from a commercial humidity sensor as well as the response from a commercial pressure sensor during three consecutive cycles of pumping air from the environment into and out of the vacuum chamber (**f**). Reproduced with permission from [112], Copyright Elsevier B.V., 2017.

Novoselov et al. demonstrated the first gas sensors of the FET type based on pristine graphene prepared by micromechanical cleavage of graphite at the surface of oxidized Si wafers, which exhibited a significant response to not only water molecules, but also NH_3_, NO_2_, and CO gases (Figure 7a) [57]. The gas sensor showed different responses to the gases due to the electronic properties, where NO_2_ and H_2_O act as acceptors, and NH_3_ and CO are donors. The exceptionally low-noise electronic property endows the graphene-based sensors with a high sensitivity. Then, the bandgap of graphene was tuned by exposing it to the humidity environment, which was increased with the amount of water molecules adsorbed on the graphene surface, thus the resistivity of the graphene increased with the humidity increasing (Figure 7b) [107]. Smith demonstrated a high-performance humidity sensor based on the electrical resistance change of CVD-grown single-layer graphene placed on the top SiO_2_ layer of an Si wafer (Figure 7c), which showed response and recovery times of less than 1 s due to the fast adsorption and desorption of the water molecules from the graphene surface (Figure 7d) [108]. They also revealed that the sensitivity of the resistance of a graphene patch to water vapor resulted from the interaction between the water electrostatic dipole moment and the impurity bands in the substrate according to the simulations. Popov’s study revealed that water molecules adsorbed at different defected places showed different effects on the resistance change of the graphene due to the different interaction mechanisms [109]. Adsorption at grain boundary defects is assumed to lead to an increase in film resistivity due to the donor property of water and the p-type conductivity of graphene, while adsorption at edge defects in multilayer graphene films leads to the formation of conductive chains with ionic conductivity. Son et al. found that physical defects in the graphene could hardly increase the humidity sensing performance, while the distinct changes were observed with chemical defects by controlling the thickness and the coverage area of the poly(methyl methacrylate) (PMMA) on the graphene surface [110].

**Figure 8:**
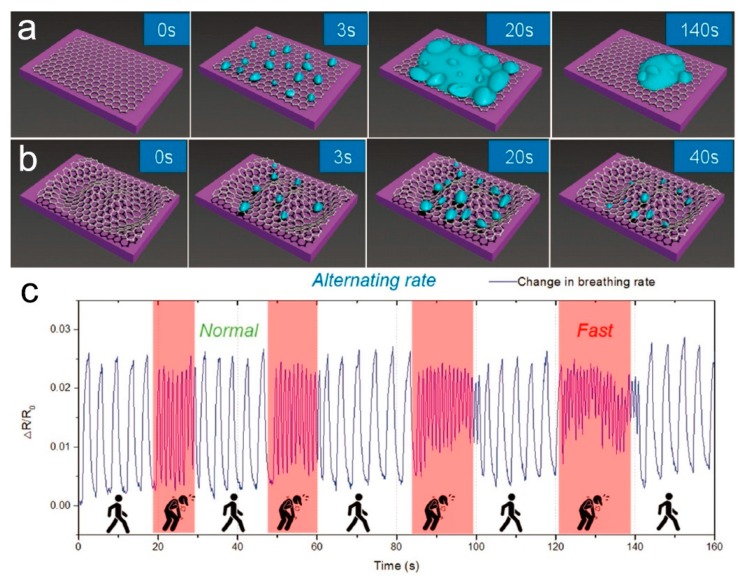
Schematic illustration of the water adsorption and desorption on surfaces of CVD-growth flat graphene (**a**) and wrinkled graphene (**b**), and breathing signals recorded by wrinkled graphene sensors with alternating breathing speed and depth during physical activity (**c**). Reproduced with permission from [113], Copyright Wiley-VCH, 2018.

Shehzad et al. demonstrated a high-performance multimode humidity sensor by constructing a graphene/Si Schottky junction. The intrinsic properties were influenced by the adsorbent water molecules. The device could detect humidity when it was both forward and reverse biased, as well as in the resistive and capacitive mode, the corresponding sensitivity of which reached 17%, 45%, 26%, and 32% per relative humidity (% RH), respectively (Figure 7e) [111]. Fan et al. investigated the gas sensing behaviors of a double-layer graphene gas sensor, the resistance of which showed a fast response and recovery towards humidity (Figure 7f), but their experiments and theoretical calculations indicated that the resistance response to the humidity of double-layer graphene was lower than that of single-layer graphene [112]. Recently, Zhu’s group reported a high-performance humidity sensor based on wrinkled graphene [113], the wrinkled morphology of which could effectively prevent the aggregation of water microdroplets and thus improve the evaporation compared to flat pristine graphene (Figure 8a,b). The device exhibited an ultrafast response to the humidity with a short response time of down to 12.5 ms, which can be used to monitor sudden changes in respiratory rate and depth (Figure 8c). Additionally, the application of graphene woven fabrics prepared with CVD growth have also been demonstrated in simultaneous sensing of humidity and temperature with high sensitivity [114]. Notably, a recent study indicated that a relative humidity of over 50% may modify the interlayer interaction, thus affecting the properties of bilayer graphene [115], therefore, the repeatability and stability of the humidity sensors based on multilayer graphene materials should be paid attention to. The sensing performance of humidity sensors based on pristine graphene materials is summarized in Table 1, which indicates that, although several humidity sensors showed a fast response and recovery, the sensitivity was relatively low and the long-term stability was not investigated.

### 4.2. Humidity Sensors Based on Graphene Oxide

**Figure 9:**
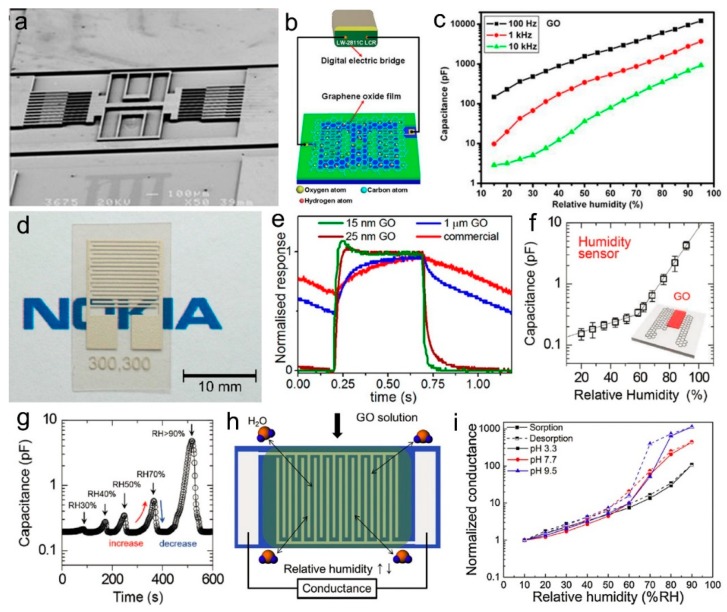
SEM image of the device (**a**), schematic diagram of the humidity testing system graphene oxide film as a humidity sensing material was placed on the two sets of interdigitated electrodes (**b**), and the output capacitances of sensors as a function of RH (**c**). Adapted from [76]. Photograph of a sprayed GO sensing element (**d**), and normalized response of the different sensors to a modulated humid air flow at 1 Hz (**e**). Reproduced with permission from [74], Copyright American Chemical Society, 2013. GO-based humidity sensor performance (capacitance vs RH) (**f**) and real-time RH sensing of the GO-based humidity sensor at specific RH (**g**). Reproduced with permission from [118], Copyright Wiley-VCH, 2016. The humidity sensing layer through drop-casting of GO followed by the measurement of relative humidity via conductance (**h**) and normalized conductance of three different humidity sensors as a function of RH (**i**). Reproduced with permission from [119], Copyright Elsevier B.V., 2017.

Humidity sensors based on GO materials have been widely studied due to the simple, low-cost, and large-scale preparation of GO, and high proton-conductive sensitivity to water molecules. Based on the change of the proton conductivity, GO humidity sensors generally operate by detecting the capacitance or impedance signals [116,117]. Bi et al. constructed a GO humidity sensor by depositing a GO film on microscale interdigitated electrodes (Figure 9a), the capacitance change of which towards humidity was measured with an LCR meter (Figure 9b) [76]. The capacitance of the humidity sensor was related with the relative humidity in the gas environment, as well as the frequency (Figure 9c). This device exhibited a highly improved sensitivity at 15–95% compared to conventional capacitive humidity sensors, and a fast response time of 10.5 s and recovery time of 41 s. Subsequently, Borini et al. investigated the influence of the GO layer thickness on the response of the humidity sensor and demonstrated that a thickness of 15 nm of the GO film resulted in an ultrafast response to a modulated humid flow at the tens-of-microseconds scale while maintaining a full scale output of over an order of magnitude (Figure 9d,e) [76]. Ho et al. reported a stretchable and multimodal all graphene electronic skin by spray coating rGO or GO on a patterned CVD graphene on poly(dimethylsiloxane) (PDMS) to realize different functions, where the device made of GO on the graphene structure worked in a capacitive mode and showed a response to the humidity change (Figure 9f,g) [118]. Recently, Park et al. investigated the correlation between the sensitivity and the sorption/desorption hysteresis of thin film GO humidity sensors working in the conductance mode (Figure 9h), which indicated that the sensors made at pH 3.3 showed a lower sensitivity and hysteresis-induced error while those made at pH 9.5 showed both increased sensitivity and hysteresis-induced error (Figure 9i) [119]. They proposed that the enhanced sensitivity and the hysteresis of these sensors were based on the molecular interactions between the increased water and charged groups in GO at high pH, suggesting a trade-off relationship between sensitivity and hysteresis. In addition, various approaches have been employed to improve the sensing performance or application area of GO based humidity sensors, such as free-standing GO foam to increase the active site [120], ultralarge GO to improve the overall proton conductivity [121], silk fiber coated with GO to take advantage of silk fiber’s flexibility [122], computer-aided design [123], investigation of the influence of the structure and coating methods [124], heteroatom-doping on GO to improve the response [125], as well as microstructure related synergic sensing for high-performance humidity sensors [126].

**Figure 10:**
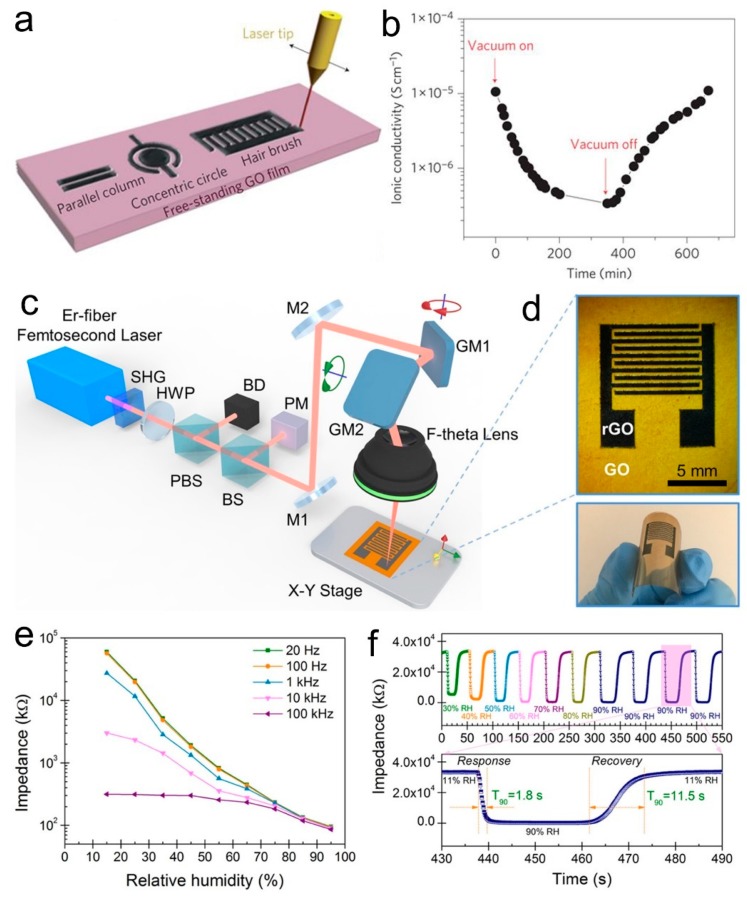
Schematic of CO_2_ laser-patterning of free-standing hydrated GO films to fabricate rGO–GO–rGO devices with in-plane geometry (**a**) and the dependence of ionic conductivity on the exposure time to vacuum and air (**b**). Reproduced with permission from [75], Copyright Macmillan Publishers Limited, 2011. Schematic image of the laser direct writing system for the single-step fabrication of all-graphene noncontact sensors (**c**), optical images of the sensor where the rGO electrodes appear in black and the brown thin film corresponds to the GO sensing material (**d**), plots of impedance as a function of RH at different operation frequencies (**e**), and (**f**) upper panel: real-time moisture sensing with different RH ranges (all starting from 11% RH) and repeated RH detection between 11% RH and 90% RH for four cycles, and lower panel: response−recovery curve of the sensor with RH switching between 11% and 90%. Reproduced with permission from [135], Copyright American Chemical Society, 2017.

Generally, noble metal (Au, Ag, etc.) interdigitated patterns prepared by the lithographical technique are used as electrodes to form GO based humidity sensors. However, Au and Ag materials are relatively expensive, and the lithographical preparation needs professional instruments and a complex process. The laser direct writing technique, which is a noncontact, fast, single-step fabrication technique with no need for masks, postprocessing, or a complex clean room, and is compatible with current electronic product lines for commercial use, has been employed to fabricate energy storage devices, electrically conductive circuits, sensors, as well as self-powered integrated devices [127,128,129,130,131,132,133]. Graphene oxide can easily be reduced by laser irradiation, producing rGO with improved conductivity, which acts as conductive electrodes, while the GO can work as a water-sensitive solid electrolyte [134]. Ajayan’s group prepared an interdigitated micro- supercapacitor (MSC) on a hydrated GO film (Figure 10a), and demonstrated that the proton conductivity of GO was related to the water concentration in the environment (Figure 10b). An et al. prepared a highly flexible humidity sensor based on an rGO/GO/rGO structure patterned by a fiber femtosecond laser (Figure 10c), which could reduce the GO to rGO with program-controlled patterns (Figure 10d) [135]. The impedance of the humidity sensor showed changes towards the relative humidity at different frequencies in a large range, indicating a high sensitivity (Figure 10e). Moreover, this device also exhibited a fast response time of 1.8 s and recovery time of 11.5 s (Figure 10f). Interestingly, this humidity sensor could be arranged in the substrate forming pixels, which could work as a noncontact e-skin with a high-spatial-resolution sensing capability.

**Figure 11:**
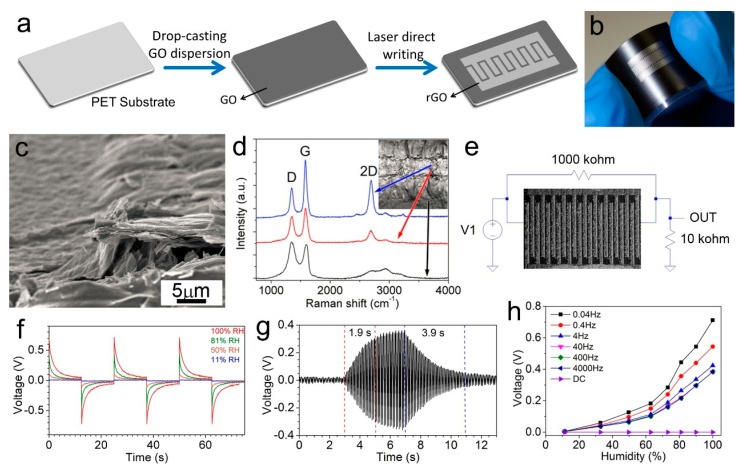
Schematic illustration for the preparation of rGO patterns by laser direct writing (**a**), interdigitated pattern of rGO/GO/rGO prepared on a flexible poly(ethylene terephthalate) (PET) film (**b**), SEM image of the structures obtained by laser direct writing (**c**), Raman spectra at different positions of a laser-irradiated line (**d**), electronic circuit of the rGO/GO/rGO humidity sensor (**e**), output waves of the humidity sensor responding to a rectangular alternating current (ac) wave with a (peak) pk−pk voltage of 1 V at 0.04 Hz (**f**), response and recovery of the humidity sensor at 40 Hz (**g**), and the change of the sensing peak voltages toward RH at different frequencies (**h**). Reproduced with permission from [131], Copyright American Chemical Society, 2018.

Recently, we reported the facile preparation of humidity sensors based on an rGO/GO/rGO structure on a flexible PET sheet through laser direct writing with a semiconductor diode laser (Figure 11a) [131]. After laser irradiation, the color of GO was turned to grey from black (Figure 11b), and the laser irradiated part expanded due to the evolved gases induced by laser heating (Figure 11c). Raman spectra, X-ray diffraction (XRD), and X-ray photoelectron spectroscopy (XPS) characterizations clearly showed that the GO was reduced to rGO after laser irradiation and became electrically conductive (Figure 11d). Instead of measuring the impedance or resistance of the rGO/GO/rGO structure, we proposed a novel alternating current (ac) detection mode by connecting the interdigitated structure to a simple circuit (Figure 11e), which showed an obvious response to the relative humidity (RH), ranging from 6.3% RH to 100% RH (Figure 11f). Compared to the low and unstable response in the direct current (dc) mode, the sensor working in this ac detection mode exhibited a dramatically enhanced sensitivity by about 45 times (Figure 11h). Moreover, the device showed a fast response time (1.9 s) and recovery time (3.9 s) (Figure 11g). The sensor also exhibited outstanding cycling stability, flexibility, and long-term stability (>1 year), as well as good reproducibility of the device preparation. The sensor could work on an iPhone to conduct the humidity sensing with an oscilloscope application on iPhone OS (iOS). Furthermore, an electronic circuit simulation method was used to fit the output waves, showing potential promise in real-time monitoring on a smartphone based on the Internet of things and big data technologies. The sensing performance of GO-based humidity sensors is summarized in Table 2, which suggests that such types of humidity sensors generally show a high sensitivity and fast response, as well as potential long-term stability. However, such types of humidity sensors are generally based on changes of the proton conductivity when adsorbing or desorbing water molecules, thus the selectivity may be disturbed by the gases of proton donors or acceptors.

### 4.3. Humidity Sensors Based on Reduced Graphene Oxide

After reduction, the insulating graphene oxide is converted into conductive rGO, the conductivity of which is sensitive to water molecules due to the defects and remnant oxygen-containing groups, and rGO also shows some capacitive behavior because of the uncomplete reduction. Till now, rGO materials prepared with various reduction methods have been employed for humidity sensing. For example, Guo et al. realized the simultaneous reduction, patterning, and nanostructuring of graphene oxide on flexible PET substrates with a two-beam-laser interference method [136]. The as-prepared humidity sensor exhibited a high sensitivity and fast response and recovery performance due to the improved water molecules’ adsorption provided by laser induced hierarchical graphene nanostructures. Humidity sensors prepared by layer-by-layer covalent anchoring of a GO film followed by a partially reduced process exhibited good sensitivity in the range of 30% to 90% RH with negligible hysteresis (<2.5% RH), short response time of 28 s and recovery time of 48 s, and good long-term stability [137]. Phan et al. investigated the influence of the reduction degree or the quantity of oxygen functional groups of the GO on humidity sensing using rapid thermal annealing [138]. They indicated that, as the annealing temperature increased, the resistivity decreased, and the GO film lost its capability to adsorb water molecules, thus the response of the humidity sensor decreased. However, a trade-off existed between the response and the long-term stability, which was quite poor in the as-deposited GO film. Recently, Shojaee et al. reported their study on the influence of the reduction degree of GO nanosheets on the humidity sensing performance by a hydrothermal reduction method, the reaction time of which accounted for the reduction degree [139]. The found that humidity sensors based on rGO with a moderate reduction exhibited an optimized sensitivity and response, because the sensitivity was attributed to the oxygen function groups while the response was attributed to the restoration of the sp^2^ carbon network. A transparent humidity sensor made of rGO stripes driven by convective self-assembly exhibited a reversible response to humidity in the range of 10–70% RH [140]. In addition, rGO materials obtained by photo reduction, such as sunlight or flash, have also been applied in high-performance rGO-based humidity sensors [141,142]. Papazoglou et al. demonstrated an in-situ sequential laser transfer and laser reduction method to fabricate rGO-based humidity sensors using picosecond laser pulses, and this laser printed rGO humidity sensor showed a fast response time of less than 1 min in the water concentration of 1700–20,000 ppm with a limit of detection of 1700 ppm [143].

Because of the importance of humidity sensing in daily life, wearable or portable humidity sensors based on rGO materials have attracted much research attention. To make the device wearable, one of the strategies is to prepare the humidity sensor in a fiber form. For example, Qu’s group demonstrated multi-stimuli sensitive sensor based on double-helix core-sheath rGO-based microfibers, which showed a high current response to small perturbations induced by temperature variations, mechanical interactions, and relative humidity changes [144]. Recently, Choi et al. developed a unique humidity sensing layer with nitrogen-doped rGO fibers on colorless polyimide film, and tiny Pt nanoparticles were deposited on the surface of rGO, which acted as dissociation catalysts for humidity sensing [145]. The rGO fiber could detect a wide humidity range from 6.1% RH to 66.4% RH, with a 1.36-fold sensitivity at 66.4% RH of pure rGO fiber. Natural fibers, such as silk fibers or spider silk fibers, have been employed as supports to construct wearable devices due to their excellent mechanical properties, superior skin affinity, and biodegradable properties. Recently, Li et al. developed a flexible humidity sensor based on silk fabrics coated with Ni and GO nanosheets, which showed a fast response to the humidity change and could be used for human respiration monitoring [146]. Li et al. demonstrated a biomimetic electric multi stimuli sensing device by combining layer-by-layer deposition of graphene and supercontraction of spidroin fibers, which exhibited a rapid response and repeatability towards RH > 20% [147]. Ma et al. proposed a strong, tough, lightweight printable biopapers by introducing silk interlayers into graphene oxide films combining a seriography-guided reduction technique, and the resultant rGO/silk patterns could work as resistive moisture sensors to detect humidity within 3.0 s, showing potential for applications in wearable electronics [148].

**Figure 12:**
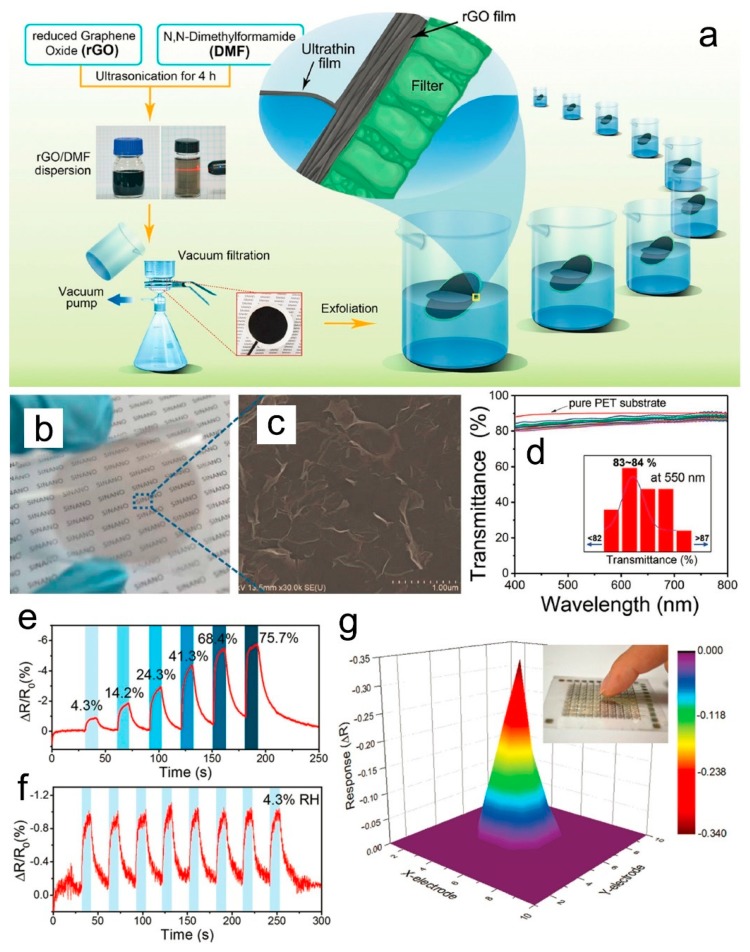
Schematic of the reproducible exfoliation process for the fabrication of r-GO ultrathin films (**a**), a typical photograph (**b**) and SEM image of an r-GO ultrathin film on a PET substrate (**c**), transmittance spectra of 13 r-GO ultrathin films on PET substrates (**d**), real-time response of one sensor in the flexible matrix device to RH from 4.3% to 75.7% (**e**), real-time-repeated response of the sensor at 4.3% RH for eight cycles (**f**), and the 3D mapping of matrix device when the fingertip approaches the relative center area of the device (**g**). Reproduced with permission from [149], Copyright Wiley-VCH, 2014.

The controllable preparation of homogeneous rGO thin film at a high efficiency is the basis for practical applications. Zhang’s group proposed an effective and reproducible way to assemble rGO ultrathin films with a controllable thickness [149]. The preparation involved the vacuum filtration of rGO suspension and exfoliation at the liquid/air interface (Figure 12a). The ultrathin film on a PET substrate showed high transparency and flexibility with layer structured rGO (Figure 12b,c). The preparation showed a good reproducibility according to the transmittance of all 13 samples (Figure 12d). The device fabricated based on rGO ultrathin films and the Au electrode showed a fast response to RH from 4.3% to 75.7% with repeated response (Figure 12e,f). Furthermore, a flexible matrix panel was prepared based on the rGO ultrathin film and Au electrode arrays, which exhibited excellent noncontact humidity sensing performance with high sensitivity, high spatial resolution, and fast response (Figure 12g). Additionally, wearable humidity sensors can also be prepared based on a porous graphene network with a high response for detecting finger humidity, speaking, whistle rhythm, and respiration monitoring [150]. The sensing performance of rGO-based humidity sensors is summarized in Table 3, which indicates that rGO-based humidity sensors generally work based on impedance or resistance change, and generally the sensitivity is not so high because of the reduced functional oxygen-containing groups compared to GO. Therefore, combining rGO and other sensitive materials may be an effective way to improve the sensitivity.

### 4.4. Humidity Sensors Based on Graphene Quantum Dots

**Figure 13:**
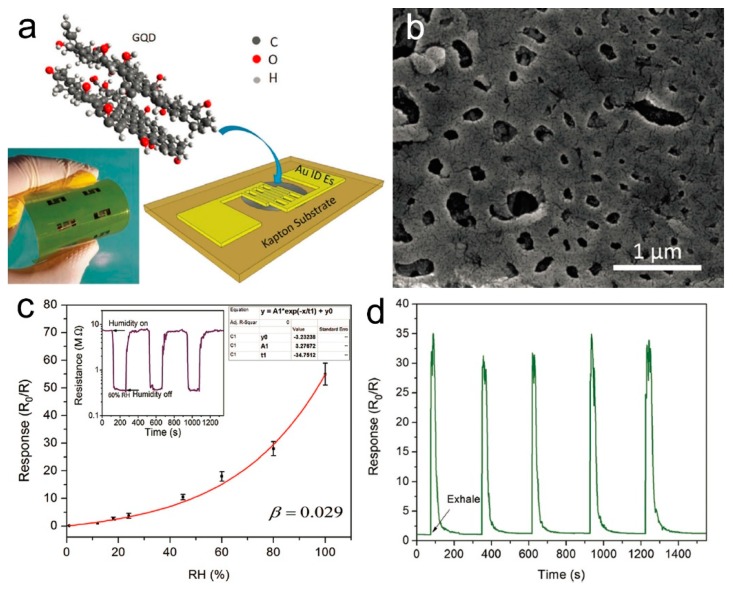
Schematic illustration of the fabricated flexible sensor (inset is an optical image of a flexible array of sensors) (**a**), SEM image of the drop-casted sensing layer (**b**), the sensor response to different levels of humidity (inset shows sensor resistance as a function of time upon exposure to 60% RH during subsequent cycles) (**c**), and response of the fabricated sensor to human breath (**d**). Reproduced with permission from [151], Copyright The Royal Society of Chemistry, 2017.

Graphene quantum dots (GQDs) have similar structures and properties as layered graphene in the 2D layer, but their electronic structure is governed by the edge electronic states and size (quantum confinement), which can be manipulated to control their properties. Sreeprasad et al. demonstrated the electron-tunneling modulation in a percolating network of graphene quantum dots selectively assembled on a polyelectrolyte microfiber, which was employed to construct a humidity sensor, where the water-mass transferred from the polymer and electrons transported through the GQDs [153]. The reduction of 0.36 nm in the tunneling barrier width between GQDs increased the conductivity of the device by 43-fold. Ruiz et al. synthesized GQDs through pyrolysis of citric acid drop-casted on a metallic interdigitated microelectrode, forming a resistive humidity sensor [152]. The sensor showed an exponential dependence of sensitivity with the RH in the range of 15–80%, and a fast response time estimated at around 5 s. The capillary condensation of water molecules on the GQD surfaces accounted for the sensing performance. Then, Alizadeh et al. prepared a GQD humidity sensor by using a similar approach [151], which exhibited outstanding sensitivity to the variation of environment humidity with a response time of about 10 s. However, they found there were two different sensing mechanisms existing between 0–52% and 52–97%. In the low RH range, the adsorption of water molecules increased the hole-type carriers, thus decreasing the electrical resistance, while in the high RH range, the improved ionic proton transportation devoted by the adsorbed water molecules induced the resistance decrease of the sensor. Hosseini et al. demonstrated the first flexible humidity sensor based on GQDs, which were prepared using a facile hydrothermal method. The as-prepared GQDs were drop-casted on an interdigitated microelectrode on a flexible polyimide substrate (Figure 13a), and the GQD film showed a porous structure (Figure 13b). The sensor showed an exponential behavior response towards humidity in the RH range of 12–100%, with a response and recovery time of 12 and 43 s, respectively (Figure 13c). The device exhibited a fast response when exposed to a flow of exhaled breath with about 90% RH (Figure 13d) [151]. From the performance summary in Table 3, it can be found that GQDs-based humidity sensors have some advantages, such as high response, high selectivity, and relatively fast response and recovery. More studies should be done to develop high-performance humidity sensors based on GQDs.

### 4.5. Humidity Sensors Based on Chemical Modified Graphene

Generally, pristine graphene or reduced graphene oxide without proper modification exhibits a relatively slow response and low sensitivity towards humidity changes, probably due to the small change in resistance, the decrease of oxygen-containing functional groups, or the aggregation during the chemical reduction process. Hence, chemical modification on graphene surfaces provides a pathway to improve the humidity sensing performance. Earlier, Huang et al. demonstrated the adding sugar through the solvothermal method could effectively tune the mount of the oxygenated group on graphene surfaces, which contributed to the improvement of the humidity sensing performance [154]. A humidity sensor based on amine rich polyethyleneimine (PEI) functionalized CVD-growth graphene showed an improved sensitivity, fast recovery, and good repeatability due to the electron transfer from amine groups in the polymer to graphene [155]. Chen et al. reported an ultra-strong PEI-GO nanocomposite by using PEI modified GO and glycerol diglycidyl ether, forming cross-linking networks [156]. Synergistic reinforcement of mechanical interlocking and hydrogen bonding led to a dramatic increase in the tensile strength and Young’s modulus by 98.3% and 87%, respectively, at 7.5 wt% GO loading of PEI and the composite film showed robust humidity sensing performance over the RH range of 40–90%. Both Su’s group and Lee’s group studied amine-modified graphene oxide as sensing materials in humidity sensors, and demonstrated that the amine modification could improve the sensitivity towards humidity change, but may bring hysteresis-induced errors due to the interaction between water molecules and amine groups [157,158]. Wang et al. proposed supramolecularly modified graphene naphthalene-1-sulfonic acid sodium salt and silver nanoparticles (Ag-NA-rGO), and the resulting supramolecular composite-based humidity sensor exhibited an excellent sensing performance between 11% and 95%, including an ultrafast response and recovery time of <1 s, and a high sensitivity and stability, which was attributed to the large surface area and wide interlayer spacing in the supramolecular composite [159]. Some chemical modification of porous graphene oxide (pGO), such as phenyl, dodecyl, or ethanol, can decrease the humidity sensing sensitivity, but improve the sensitivity to other molecules [160].

**Figure 14:**
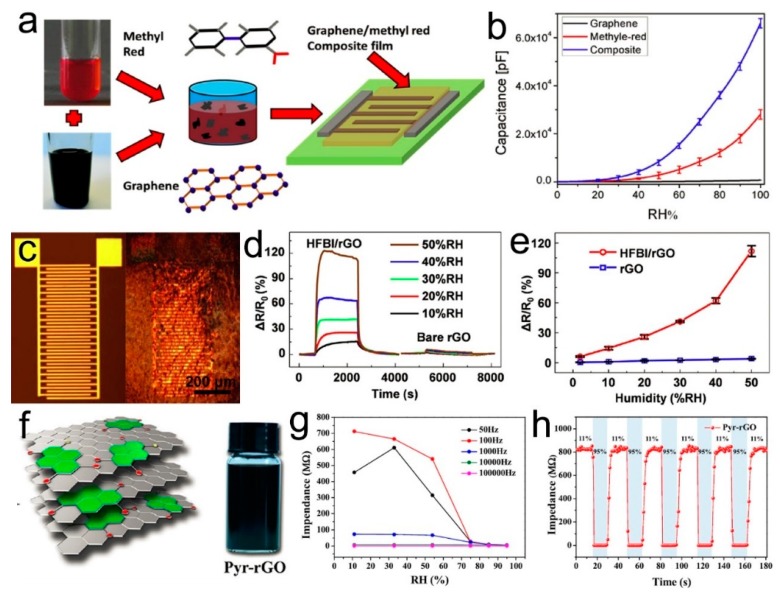
Schematic representation of the graphene/methyl-red composite based humidity sensor (**a**), and capacitance versus relative humidity (% RH) characteristics curves of the graphene/methyl-red composite, methyl-red only, and graphene only based humidity sensors measured in the humidity chamber at a 1 kHz frequency (**b**). Reproduced with permission from [161], Copyright, 2016 Elsevier B.V. Optical images of the comb electrode before and after coating a layer of hydrophobin (HFBI) wrapped rGO flakes (**c**), real-time responses of HFBI wrapped rGO sensor (left) and bare rGO sensor (right) to RH ranging from 2% to 50%, respectively (**d**), and relative resistance change upon the exposure to water molecules at the maximum equilibrium vs. RH values (**e**). Reproduced with permission from [162], Copyright Elsevier B.V., 2017. Schematic of the supramolecular assembly of Pyr-rGO sheets with the corresponding physical image in dispersion (**f**), the impedance curves of Pyr-rGO based humidity sensors measured at different frequencies under different RH levels (**g**), and the five-cycle response-recovery curve of Pyr-rGO (**h**). Adapted from [163].

In order to address the low resistance change of graphene towards humidity, Ali et al. introduced methyl red molecules to modify graphene surfaces and the composite was deposited over the interdigitated electrodes with the electrohydrodynamic technique (Figure 14a). The electrical resistance of the humidity sensor varied inversely over a wide range from 11 to 0.4 MΩ towards the RH content from 5% to 95%, and the humidity sensor showed 96.36% resistive and 2,869,500% capacitive sensitivity (Figure 14b), with a fast response and recovery times of 0.251 and 0.35 s, respectively [161]. The highly-improved humidity sensing performance was attributed to the water adsorption on methyl red, and induced a decrease of the overall film resistance and the paths between graphene flakes built by water adsorbed methyl red. Tao et al. proposed the preparation of hydrophobin (HFBI) protein wrapped rGO by a one-step exfoliation and functionalization, which was casted onto the interdigitated electrode (Figure 14c) [162]. Humidity sensors based on HFBI modified rGO showed a highly improved sensitivity compared to that based on pristine rGO (Figure 14d,e). Chen et al. employed a similar supramolecular assembly method to modify rGO with functional organic molecule pyranine, which has a pyrene ring decorated with hydrophilic sulfonic groups and can assemble with rGO through π-π interactions (Figure 14f) [163]. A humidity sensor based on this composite exhibited an excellent sensing performance, including a high sensitivity between 11% and 95% RH, fast response time of <2 s, small hysteresis of 8% RH, and good repeatability and stability (Figure 14g,h). Additionally, tannic acid modified rGO can be incorporated in PVA to improve its humidity sensing properties [164]. Recently, a typical metal organic framework (MOF), copper benzene-1,3,5-tricarboxylate (Cu-BTC), was incorporated and deposited on GO film to form a resistive humidity sensor, which showed an improved humidity sensing performance due to the facilitated water molecules captured by Cu-BTC from the environment [165]. The sensing performances of humidity sensors based on chemical modified graphene are summarized in Table 4, wherein humidity sensors based on graphene/methyl-red or pyranine-rGO showed excellent performance, but further studies on the stability and selectivity are required.

### 4.6. Humidity Sensors Based on Graphene/Polymer Composites

It is well-known that electric humidity sensors output electrical signals by sensing the variation of the moisture content by adsorbing/desorbing water molecules in the environment on the sensitive layer. To construct humidity sensors, polymers are one of the chosen sensitive materials, because they have some advantages, such as low cost, good sensitivity, fast response, flexibility, easy processability, etc. However, generally, they lack conductivity and show large hysteresis due to the cluster of water adsorbed inside bulk polymers, which may cause deformation and instability of the sensing polymer layer, eventually reducing the lifetime of the sensor. Therefore, the combination of polymers and graphene can harness the advantages of both to improve the humidity sensing performance and this has been widely investigated.

Cellulose is an abundant, renewable, and natural material on earth, which is a colorless, odorless, and nontoxic solid polymer, and possesses the advantages of a high mechanical strength, hydrophilicity, relative thermo-stability, biocompatibility, light weight, low price, and is eco-friendly. Moreover, cellulose is a high dielectric material and insulator, therefore, the incorporation of cellulose into graphene may be of benefit to the humidity sensing performance. For example, Kim’s group first employed cellulose nanocrystals to modify rGO, and the resultant composite, m-r(CNC/GO), exhibited an improved sensitivity by 5 times compared to that of pristine rGO, which was attributed to its high surface-to-volume ratio and charge-storage capacity at junctions related to hydrophilic functional groups, such as carboxyl groups [166]. Then, they demonstrated that the incorporation of GO can highly improve the humidity sensor based on cellulose nanocrystal (CNC) due to the increased water molecule adsorption; the water uptake of the cellulose/GO composite in 90% RH was almost two times higher than the pristine cellulose film [167]. Nanocellulose was also used to assist graphene dispersion in the PVA nanocomposite (PVA/NFC/rGO) for humidity sensing, and the abundant hydroxyl groups on the surface of nanocellulose formed hydrogen bonds with water molecules for an improved sensitivity and decreased hysteresis [168]. Recently, Chen et al. prepared a cellulose/graphene composite film using an eco-friendly process by dispersing GO and cellulose homogeneously followed by in situ chemical reduction of GO to rGO, endowing the film with high conductivity and good mechanical properties [169]. The composite film was used as a sensing material to different external stimuli, such as temperature, stress/strain, liquids, as well as humidity in a resistance change. The swelling of the cellulose matrix by adsorbing water molecules increased the distance between the rGO sheets, thus increasing the resistivity of the material.

**Figure 15:**
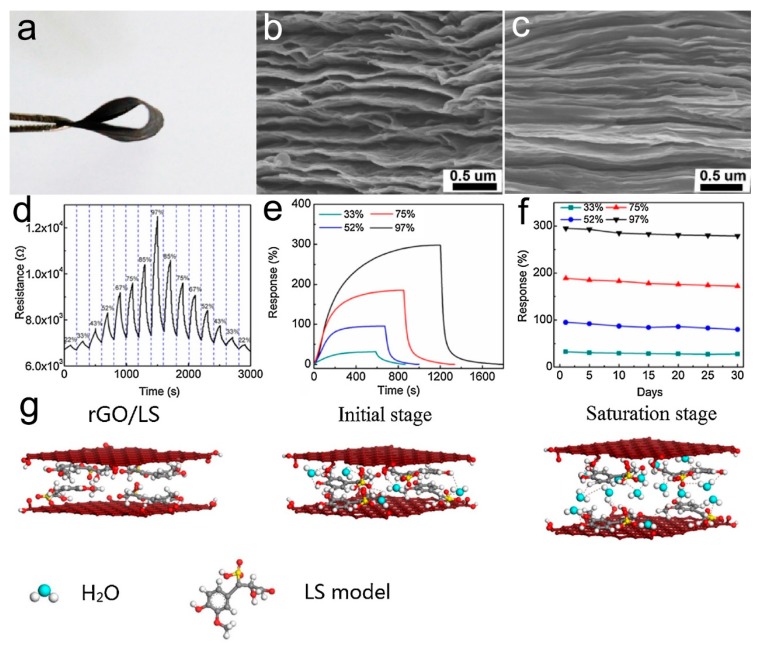
Photographs of a flexible rGO/LS-1 thin film (**a**), cross-sectional SEM images of rGO (**b**) and rGO/LS-1 (**c**) thin-film, real-time resistance measurement of the rGO/LS thin-films under switching RH (**d**), time-dependent humidification-dehumidification curves to a relative humidity pulse between 0% and 33%, 52%, 75%, and 97%, respectively (**e**), long-term stability of rGO/LS thin-film at 33%, 52%, 75%, and 97% RH (**f**), and schematic image of the humidity sensing mechanism of an rGO/LS thin-film at the initial stage and the saturation stage under a humidity environment, respectively (**g**). Reproduced with permission from [170], Copyright Elsevier B.V., 2017.

Lignin is also an abundant polymer in nature and the largest biomass source with an aromatic skeleton. Technical lignin from biomass can be sulfonated into water-soluble sodium lignosulfonate (LS) with abundant oxygen containing groups, which can provide a large number of moisture acceptors for humidity sensing. The hydrophobic phenylpropane units of LS can be spontaneously cross-linked into 3D networks, which is beneficial for the fast swelling and shrinking during adsorption and desorption of water molecules. Nevertheless, LS is electrically insulating, preventing it from being applied in resistive humidity sensors. Recently, Chen et al. designed an LS/rGO composite-based resistive humidity sensor using LS as the moisture sensing layers and rGO as the resistance transduction layers [170]. The LS/rGO composite film showed good flexibility (Figure 15a) and exhibited a more compact interlayered structure compared to pure rGO film (Figure 15b,c), and this alternative multilayered structure could provide water molecules as accepted sites as well as resistant transduction. The LS/rGO humidity sensor showed an apparent response to the RH from 22% to 97% with a dependent resistance increase with the increasing RH (Figure 15d). It also showed a relatively fast response to the RH change (Figure 15e) and a long-term stability lasting for 30 days (Figure 15f). The sensing mechanism may be attributed to the reduction of the hole density in the p-type rGO layers caused by physisorbed water molecules in low humidity and the interlayer swelling effect of LS networks in high humidity (Figure 15g).

Polyelectrolytes are always employed in humidity sensors due to their function groups being sensitive to water molecules. However, their impedance at low relative humidity levels is too high to be measured exactly due to their conductivity, while graphene materials have the advantage of a high surface area and relatively high conductivity. Therefore, it is an effective strategy to improve the sensing performance by combining polyelectrolytes and graphene materials. Li et al. first prepared two composites of polyelectrolytes, i.e., cationic poly(diallydimethylammonium chloride) (PDDA) and anionic sodium poly(4-styrenesulfonate) (PSSNa), and rGO as sensing materials [171]. Both the polyelectrolytes and polyelectrolyte/rGO showed a high response towards RH in the range of 10–90%. However, at lower RH, the polyelectrolytes showed an impedance that was too high to detect. With an increase of the rGO concentration in the polyelectrolytes, the impedance at low humidity was largely decreased by ~45 times. Both composites exhibited high sensitivity and good sensing linearity in their impedance response to humidity in the RH range of 0.2–30%. Typically, PDDA/rGO showed a higher sensitivity of 1000% with respect to the 300% for PSSNa/rGO. Subsequently, they prepared another impedance-type polyelectrolyte/graphene humidity sensor by sequentially depositing the thin films of cross linked and quaternized poly(4-vinylpyridine) (QC-P4VP) and rGO onto interdigitated gold electrodes [172]. The QC-P4VP/rGO humidity sensor showed much lower impedance than the QC-P4VP humidity sensor in the low RH levels and could detect ultra-low RH of 0.18% with a high response with an increase of 500% between 1.1% and 0.18% RH. Moreover, it showed a small hysteresis of ~4.5% RH and a fast response time of 21 s and recovery time of 78 s. Zhang et al. reported rGO/PDDA and GO/PDDA composite humidity sensors by using a layer-by-layer self-assembly approach. The rGO/PDDA exhibited a resistive type humidity sensor, which showed a stable and fast response to the RH in the RH range of 11–97% with a fair sensitivity, and the sensing mechanism was attributed to the p-type semiconducting properties of rGO at low RH, and interlayer swelling of rGO/PDDA film at high RH rather than the ionic conductivity [173]. Then, they demonstrated an ultrahigh performance humidity sensor based on GO/PDDA working in a capacitive type. The humidity sensor showed an unprecedented response of up to 265,640% in the RH range of 11–97% with a short response and recovery time of within 1 s. Thus, it can be used to sense human breath. The excellent sensing performance was attributed to enhanced proton transportation and water molecule permeation in the mesoporous film with adsorbed water molecules [174].

**Figure 16:**
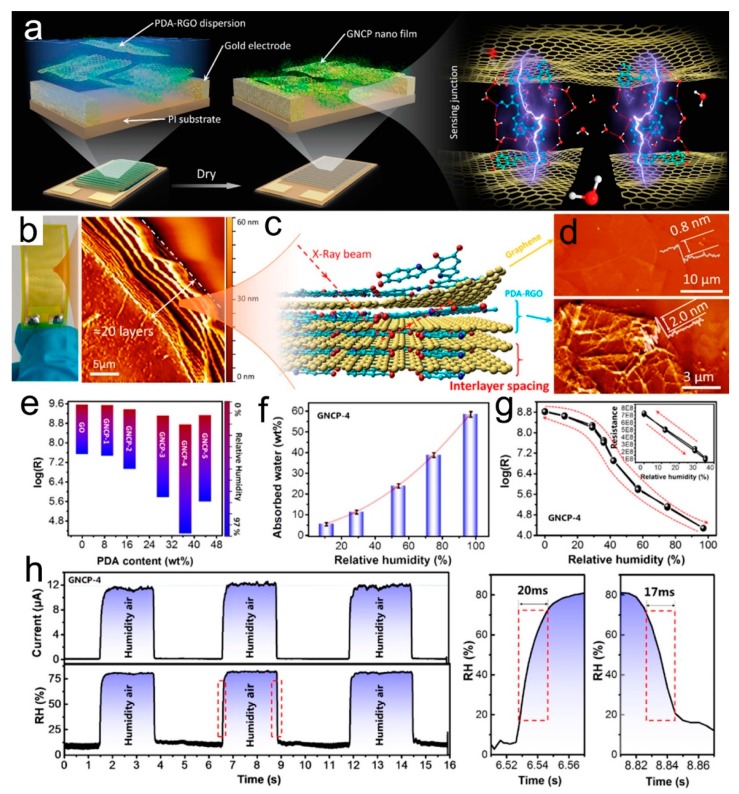
Schematic fabrication layer-by-layer stacking process of the graphene nanochannels confined poly(dopamine) (GNCP) high order superlattice sensing junction structure (**a**), photograph of a drop-casted GNCP sensing element and atomic force microscope (AFM) image of nano-size layered structure poly(dopamine) (PDA)/graphene film on it (**b**), illustration of superlattices composed of alternating atomic scale PDA/graphene layers (**c**), AFM image of graphene before (up) and after PDA modification (down) (**d**), the RH dependent resistance response range of PDA/graphene sensors as a function of PDA content (**e**), histogram plots of absorbed water of PDA/graphene at different humidity atmospheres (**f**), the derived RH dependent resistance changes of PDA/graphene and its magnified curve of the low RH region from 0% to 35% (**g**), and the changes in the measured current from the film at 1 V as RH was switched between dry air (RH ≈ 10%) and humidity air (RH ≈ 80%) (right) (**h**). Estimated results showed the ultrafast response (20 ms) and recovery (17 ms) times (left). Reproduced with permission from [176], Copyright American Chemical Society, 2018.

**Figure 17:**
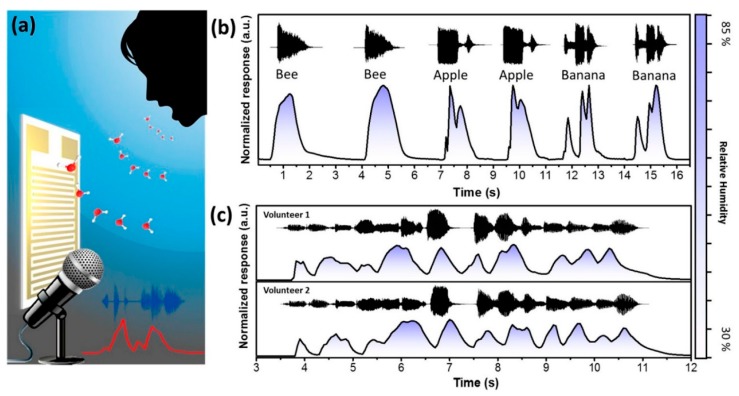
(**a**) Schematic illustration of a humidity sensor for human exhaled air detection during speaking. (**b**) Repeated responses of a PDA/graphene sensor to three different words. (**c**) Responses of a PDA/graphene sensor to the song “Twinkle Twinkle Little Star” sung by two different volunteers. Reproduced with permission from [176], Copyright American Chemical Society, 2018.

Dopamine is a small molecule containing catechol and amine groups, and its polymerized form, known as poly(dopamine) (PDA), is similar to the adhesive proteins of mussels, which can be used to modify the materials to bind strongly to most organic and inorganic surfaces. Hwang et al. demonstrated the incorporation of PDA-GO into PVA to synthesize graphene-reinforced PVA composite films, during which PDA reduced GO to rGO [175]. This film showed reinforcement and toughening mechanical properties, as well as an apparent response to RH in the range of 40–100% due to the proton generation on the surface of rGO and PVA when adsorbing water molecules. Recently, He et al. reported a high performance humidity sensor for wearable devices through a bioinspired atomic-precise tunable graphene/PDA heterogeneous sensing junction [176]. The strategy for preparing the bioinspired graphene nanochannels confined poly(dopamine) (GNCP) sensor is schematically shown in Figure 16a, which indicates the dispersion of PDA modified rGO was drop-cased on the flexible polyimide substrate with an interdigitated Au electrode. After slowly evaporating the solvent, the rGO/PDA nanosheets spontaneously self-assembled into a 40 nm thick rGO/PDA film where monolayer graphene alternated with PDA molecular layers with abundant graphene-polymer heterogeneous sensing junctions (Figure 16b–d). The humidity sensing range of the rGO/PDA depended on the PDA content (Figure 16e). The typical composite of GNCP-4 was highly sensitive to variation of RH and improved by almost 4 orders of magnitude with little hysteresis when the RH changed from 0% to 97% (Figure 16f,g). This device also showed a very fast response and recovery, with a response time of about 20 ms and recovery time of 17 ms (Figure 16h,i). A large number of hydroxyl and amino groups in the PDA polymer acting as proton donors and accepters could provide a possible proton transport mechanism by proton-hopping among the carboxyl and amino. Furthermore, this humidity sensor could detect humidity fluctuation information for voiceprint recognition anticounterfeiting (Figure 17), and monitor human respiratory, as well as be applied in noncontact human skin activity real-time monitoring devices.

In addition, some other polymers composited with graphene materials have also been reported, such as poly(N-vinyl pyrrolidone) (PVP), polypyrrole (PPy), poly(vinyl alcohol) (PVA), polyvinylidene fluoride (PVDF), polyurethane (PU), and Nafion. For example, an rGO/PVP composite based resistive humidity sensor was highly sensitive to RH in the range of 30–90% with a response time of ~3 s. The water molecules adsorbed by PVP assisted the charge transfer on the rGO/PVP composite sheets, thus increasing the sensitivity and response [177]. The PVP could also assist the dispersion of graphene to form graphene/PVP ink, which was inkjet-printed on the interdigitated electrode to form a humidity sensor, and further integrated into a complementary metal oxide semiconductor (CMOS) to fulfill the humidity sensing [178]. Su et al. prepared an rGO/PVP resistive humidity sensor, which showed high sensitivity towards RH in the range of 7–97.3% with a response and recovery time of 2.8 and 3.5 s, respectively. Interestingly, such a humidity sensor was integrated with a triboelectric nanogenerator to form a self-powered humidity sensor [179]. Hernández-Rivera et al. reported a capacitive humidity sensor based on an electrospun PVDF/graphene membrane, the sensing principle of which was based on the dielectric constant change of membranes due to the water vapor inside fibrous structure, and the enhanced response of the device may be caused by the improved hydrophobicity brought by the incorporation of graphene into the PVDF [180]. Lin et al. prepared a graphene/PPy composite by a chemical oxidative polymerization method with graphene, which acted as the sensing materials in the impedance humidity sensor. The humidity sensor based on 10% graphene/PPy material showed optimized sensing properties in the RH range of 12–90% with a higher sensitivity of 138 and small humidity hysteresis of <0.16%, as well as fast response and recovery times of 15 and 20 s, respectively [181]. Trung et al. reported a high-performance humidity sensor based on a rGO/PU composite sensing layer and an elastomeric conductive electrode, which exhibited fast response and recovery times of 3.5 and 7 s, respectively. Furthermore, this device remained almost unchanged under stretching up to a strain of 60% and after 10,000 stretching cycles at a 40% strain in the presence of humidity, meaning it can easily be attached to a finger to monitor humidity [182]. Recently, Leng demonstrated that the incorporation of Nafion into diamine modified GO (MGO) could improve the linearity of the sensor compared to MGO [183]. Additionally, fullerene and multiwalled carbon nanotubes have been introduced to composites with graphene to form humidity sensors with improved performances by increasing the accessible surface area of the composite material and accelerating the diffusion of the water molecules [184,185]. Plenty of humidity sensors based on graphene/polymer composites have been reported, the performances of which are summarized in Table 5. The combination with polymer can endow the composite with not only improved sensing performances, such as higher sensitivity and lower detection limit, but also additional functions, such as strength, flexibility, adhesion, and stability. Nevertheless, as we all know, the aging of polymers will be an issue for polymer-incorporated humidity sensors during their practical use. Therefore, accelerated aging tests on humidity sensors based on graphene/polymer composites are suggested.

### 4.7. Humidity Sensors Based on Graphene/Metal or Metal Oxide Composites

**Figure 18:**
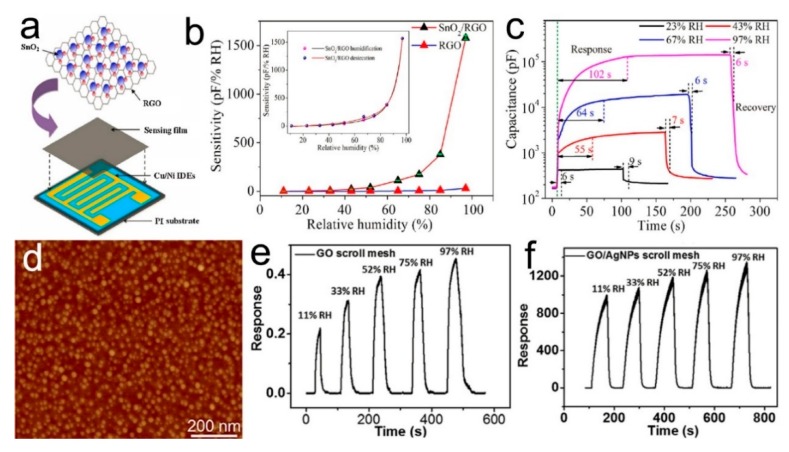
Schematic illustration of as-fabricated sensor prototype (**a**), sensitivity comparison between SnO_2_/rGO composite and rGO towards humidity (**b**), and response and recovery curves of the SnO_2_/rGO composite sensor towards an RH pulse from dry air to other RH levels (**c**). Reproduced with permission from [77], Copyright Elsevier B.V., 2015. AFM images of GO-Ag sheets (**d**), time-dependent response and recovery curve of an rGO scroll meshes-based device (**e**) and an rGO-Ag scroll meshes-based device (**f**) at different humidity. Reproduced with permission from [195], Copyright The Royal Society of Chemistry, 2017.

Metal oxide nanostructures, such as SnO_2_, CuO, ZnO, and TiO_2_, showed humidity sensing performance due to their high specific surface area, diverse morphology, vacancies, and defects, but a lack of conductivity and slow electron diffusion limited the response towards humidity while pristine GO or rGO exhibited relatively poor sensing properties toward humidity, including low sensitivity and irreversibility. Thus the composite of graphene and metal oxide structures may improve the performance of the humidity sensor. Graphene/SnO_2_ composite was widely studied in humidity sensing, and the incorporation of graphene could highly improve the humidity sensing performance. For example, graphene coated SnO*_x_*/carbon fiber (graphene/SnO*_x_*/CF) showed a sensitivity of 6.22, more than 2 times more than the 2.71 for the uncoated one [186]. Xu et al. demonstrated a humidity sensor based on GO wrapped SnO_2_@graphene, which showed a very high sensitivity of up to 32 MΩ/% RH, fast response and recovery time of <1 s, and good stability, which could be attributed to the improved conductivity by graphene and oxygen-rich groups (hydroxyl and epoxy groups) from GO [187]. Zhang et al. fabricated SnO_2_ nanoparticles/rGO composites by using a facile one-step hydrothermal route, and deposited the composite on microelectrodes forming the humidity sensor (Figure 18a) [77]. The device showed highly improved sensitivity compared to the pristine rGO (Figure 18b), as well as fast response and recovery (Figure 18c), which may be attributed to the active sites, such as vacancies and defects, brought by SnO_2_ nanoparticles and the heterojunction created at the interface of the two materials. They also investigated the humidity sensing performance of the SnO_2_ nanoparticles/rGO based humidity sensor working in a resistive mode [188]. Reduced GO was also employed to improve the sensing performance of the humidity sensor based on Fe-doped SnO_2_ [189]. For other metal oxide/graphene composites, Wang et al. demonstrated that a humidity sensor based on rGO/CuO composite exhibited a relatively good humidity sensing performance, including high sensitivity and fast response, which was attributed to the formation of an rGO−CuO Schottky junction [190]. It has also been demonstrated that the incorporation of graphene shows improvements on the sensing performance of humidity sensors based on ZnO or TiO_2_ nanoparticles [191,192,193,194].

Several works have studied the combination of metal nanoparticles and graphene applied in humidity sensors. For example, Liu et al. prepared Ag nanoparticles encapsulated GO scrolls to form GO-Ag scrolls by a molecular combing method, where Ag nanoparticles uniformly deposited on the GO layer (Figure 18d) [195]. After reduction by hydrazine, the humidity sensor based on rGO-Ag scroll meshes exhibited a 3 orders of magnitude response towards humidity compared to that of rGO scroll meshes (Figure 18e,f). The excellent sensitivity was attributed to the enhanced conductivity of rGO-Ag scroll meshes induced by the encapsulation of Ag nanoparticles. Su et al. demonstrated the self-assembly of Au nanoparticles on the surface provided conduction pathways, thus improving the sensitivity and linearity of the sensing film [196]. Interestingly, Yeo et al. realized the suppression of humidity dependence of the rGO sensor by incorporating Cu nanoparticles to decrease the electrical resistances to detect other gases [197]. The sensing performance of humidity sensors based on graphene/metal oxide or metal nanoparticles is summarized in Table 6.

### 4.8. Humidity Sensors Based on Graphene/2D Materials

**Figure 19:**
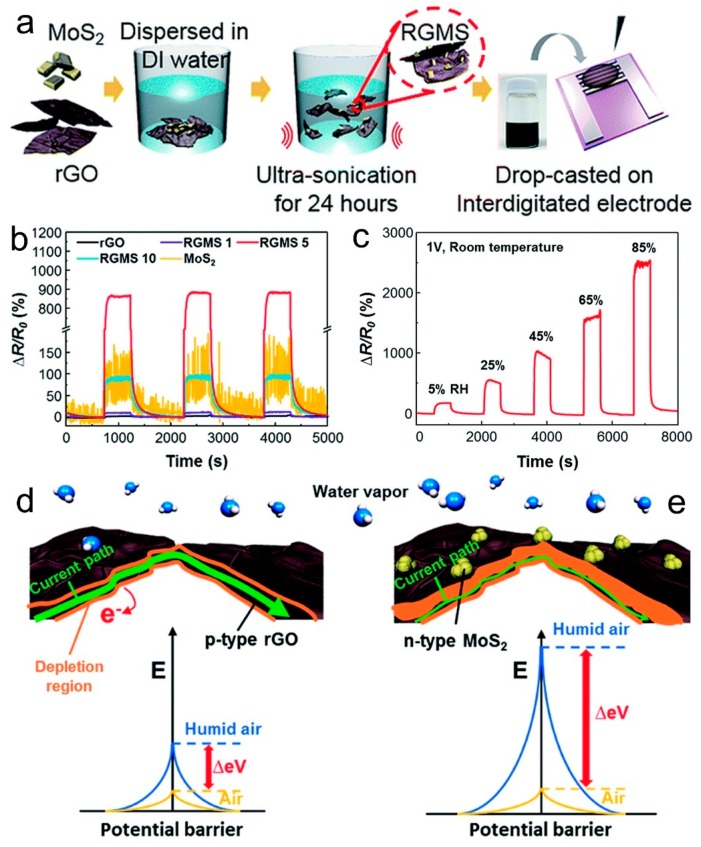
Fabrication procedure of the rGO@MoS_2_ humidity sensor (**a**), response curves of rGO, RGMS 1, RGMS 5, RGMS 10, and MoS_2_ to 50% RH at 25 °C (**b**), response curves to different RHs of 5%, 25%, 45%, 65%, and 85% at 1 V (**c**), and Schematic of the mechanism with enhanced humidity sensing properties of the enhanced depletion region on (**d**) bare rGO and (**e**) RGMS. Reproduced from [199] with permission from The Royal Society of Chemistry.

Recently, other 2D materials, such as transition metal dichalcogenides, like MoS_2_ and WS_2_ and black phosphorus, have attracted increasing attention for ultrasensitive sensor applications due to their unique structure and electronic properties. Several 2D materials, such as MoS_2_, WS_2_, and black phosphorus (BP), have been employed for composites with graphene as humidity sensing materials for humidity sensors with improved performance. Burman et al. developed the first MoS_2_/GO nanocomposite-based humidity sensor, which exhibited a high sensing response lying between 55 times at 35% RH and 1600 times at 85% RH, and the high response was attributed to the high proton conductivity in the water layer for both MoS_2_ and GO [198]. Recently, Park et al. reported chemoresistive humidity sensors based on rGO/MoS_2_ composites, which were prepared by simple ultrasonication without additives and additional heating followed by drop-casting on the interdigitated electrodes (Figure 19a) [199]. The rGO/MoS_2_ humidity sensor exhibited a 200 times higher response to humidity at room temperature, compared to the pristine rGO humidity sensor (Figure 19b), and showed a dependent response towards humidity change (Figure 19c). The electronic sensitization due to p–n heterojunction formation and porous structures between rGO and MoS_2_ accounted for the remarkable improvement in the sensing performance of the rGO/MoS_2_ humidity sensor (Figure 19d,e). They also demonstrated a high-performance humidity sensor based on rGO/MoS_2_ hybrid composites synthesized by the hydrothermal method [200]. In addition, WS_2_/GO nanohybrids humidity sensors have also been demonstrated with a high response up to 65.8 times at 40% RH and 590 times at 80% RH and a fast response time of 25 s and a recovery time of 29 s, which was attributed to the oxygen linking activities at the GO/WS_2_ interface for better proton conductivity [201]. BP materials showed ultra-sensitive humidity sensing performance due to the natural adsorption of water molecules induced by the specific 2D layer-crystalline structure, but lack repeatability due to the instability of BP with water molecules. Phan et al. introduced graphene into BP forming BP/graphene composite to overcome this limitation, and the stability of the humidity sensor was improved by the BP/graphene interface. The humidity sensor exhibited a linear response in the range of 15–70% RH with a sensitivity of 43.4% at 70% RH, and a fast response time of 9 s and a recovery time of 30 s [202]. Humidity sensors based on graphene/2D materials are listed in Table 6, which indicates that graphene/MoS_2_ composites have large potential in high-performance humidity sensors, but the response and recovery times still need to be reduced.

## 5. Summary and Perspective

Various effective approaches have been devoted to developing graphene-based humidity sensors with high sensing performance. First, humidity sensors based on pristine graphene show high sensitivity with a detecting limit as low as 1 ppm and fast response, however, it suffers from low selectivity and relatively slow recovery. Chemical modification on the graphene surface may be a possible way to improve the selectivity and recovery. Second, graphene oxide-based humidity sensors possess high sensitivity and fast response in the high humidity range, but it is difficult for them to detect low humidity conditions of less than 5% RH. The swelling effect and relatively low stability possibly exist at high RH ranges, and the capacitive or impedance working mode generally needs relatively complicated circuits. Third, reduced graphene oxide provides an effective strategy to construct resistive humidity sensors with simple and easy fabrication and measurements and low power consumption, but it also reduces the available functionional groups for adsorbing water molecules, thus decreasing the sensitivity and sensing humidity range. Fourth, various kinds of materials, such as chemicals, polymers, metal, metal oxide, and 2D materials, have been incorporated into graphene oxide or reduced graphene oxide film to improve the humidity sensing performance, and some of them show excellent performances, such as wide sensing RH range, high sensitivity, small hysteresis, and fast response, but they may suffer from reproducibility and long-term stability.

Table 7 provides the performance of several graphene-based humidity sensors with superior comprehensive characteristics. All of them have high sensitivity, fast response, and broad humidity range, but there are still some challenges in the preparation and practical applications of the graphene-based humidity sensors. First, the design and development of humidity sensors based on graphene-materials with a comprehensively high performance, including high sensitivity, high selectivity, wide humidity range, fast response and recovery, and small hysteresis, is still required, and the long-term stability must be especially addressed. Second, the reproducibility for graphene-based materials is a big challenge, because the preparation process, especially the oxidation degree and reduction degree of graphene, is quite difficult to control precisely, thus resulting in materials with different sensing performance. Third, although several studies involve detection in a low humidity range of less than 2% RH, it still requires studies addressing the sensing in the low humidity range. Fourth, besides sensing performance, it is time to consider issues involved in the commercialization of graphene-based humidity sensors, such as large-scale manufacturing, integration, encapsulation and packaging, repeatability, long-term stability in practical use, anti-scratch and anti-chemical characteristics, calibration-free characteristics, etc. Fortunately, Goldsmith et al. have demonstrated a process on industrially manufactured graphene-based digital biosensors [203], but the commercialization still has a long way to go. Furthermore, most current studies on graphene-based humidity sensors have not considered the power consumption issue, which is very important for the future of wearable electronics.

## Figures and Tables

**Table 1:** Summary of the sensing performances of humidity sensors based on pristine graphene.

Material	Preparation Method	Type	Operating Temperature	Sensitivity	Selectivity	Humidity Range	Response/Recovery Time	Stability	Ref.
Graphene	micromechanical cleavage of graphite	FET	R.T.	—	Low (NH_3_, CO, NO_2_)	1 ppm	Hundreds of seconds	—	[57]
Graphene	CVD	Resistive	R.T.	0.31%	High (Ar, N_2_, O_2_)	1–96%	0.6 s/0.4 s	—	[108]
Multilayer graphene	CVD	Resistive	25 °C	10–17%	—	15–80%	<1 s	—	[109]
Graphene/Si Schottky junction	CVD	Resistive Capacitive	10–90 °C	45% 32%	High (N_2_, O_2_, Ar, CO_2_)	10–90%	8 s/19 s 4 s/10 s	—	[111]
Double-layer graphene	CVD	Resistive	25 °C	~0.784–0.933%	Low (CO_2_)	20–100%	Hundreds of milliseconds	—	[112]
Wrinkled graphene	CVD	Resistive	20–80 °C	—	—	11–95%	12.5 ms	—	[113]

**Table 2:** Summary of the sensing performance of GO-based humidity sensors.

Material	Preparation Method	Type	Operating Temperature	Sensitivity/Response	Selectivity	Humidity Range	Response/Recovery Time	Long-Term Stability	Ref.
GO	Drop casting	Capacitive	25 °C	37,800%	—	15–95%	10.5 s/41 s	30 days	[76]
GO	Drop casting	Impedance	10–40 °C	—	—	10–90%	~30 ms	72 h	[74]
GO	Spary coating	Capacitive	22–90 °C	—	—	20–90%	—	500 cycles	[118]
GO	Drop casting	Conductance	25 °C	12.3 ± 2.2 μS/%RH (pH 3.3) 12.3 ± 2.2 μS/%RH (pH 9.5)	—	10–90%	2.2 s/1.6 s (pH 2.8) 91.8 s/11.3 s (pH 9.3)	—	[119]
GO foam	Dry in a frame	Impedance	25 °C	33,254%	—	11–95%	50 s/79 s	—	[120]
Ultralarge GO	Drop casting	Conductance	20 °C	4339 ± 433	—	7–100%	0.2s /0.7 s	5 days	[121]
GO	Simulation	Capacitive	25 °C	7680 pF/%RH	—	0–100%	<0.5 s	—	[123]
Li-doped GO	Drop casting	Resistive	25 °C	3038%	—	11–97%	4 s/25 s	—	[125]
GO	Spary coating	Capacitive	R.T.	—	—	12–97%	<0.1 s	—	[126]
rGO/GO/rGO	Laser direct writing	Impedance	23 °C	—	—	11–95%	1.8 s/11.5 s	30 days	[135]
rGO/GO/rGO	Laser direct writing	Voltage	R.T.	142.5	High (H_2_, hexane, ethanol)	6.3–100%	1.9 s/3.9 s	> 1 year	[131]

**Table 3:** Summary of the sensing performance of humidity sensors based on reduced graphene oxide or graphene quantum dots.

Material	Preparation Method	Type	Operating Temperature	Sensitivity/Response	Selectivity	Humidity Range	Response/Recovery Time	Long-Term Stability	Ref.
rGO	Two-beam-laser interference reduction	Capacitive	25 °C	—	—	11–95%	3 s/10 s	—	[136]
rGO	LBL-anchored and chemical reduction	Impedance	15–35 °C	0.0423 log Z/%RH	—	30–90%	28 s/48 s	42 days	[137]
rGO	Rapid thermal annealing	Resistive	25 °C	35.3–0.075%	High (Acetone, ethanol, toluene, NH_3_, H_2_, CO, NO_2_, C_2_H_2_)	20–95%	—	5 weeks	[138]
partially reduced GO	Hydrothermal reduction	Resistive	R.T.	3.3–105%	—	20–85%	4.2 s/3.6 s	14 days	[139]
rGO	Chemical reduction	Resistive	25 °C	17.6	—	10–70%	—	—	[140]
rGO	Sunlight reduction	Impedance	25 °C	three orders of magnitude	—	11–95%	16 s/47 s	30 days	[141]
rGO	Flash reduction	Impedance	20 °C	—	—	11–95%	1 s/24 s	30 days	[142]
N-doped rGO fiber	Thermal annealing	Resistive	R.T.	0.32–4.51%	—	6.1–99.9%	—	30 cycles	[145]
rGO-silk	Seriography-guided reduction	Resistive	25 °C	—	—	~20–97%	3 s/~1 min	—	[148]
rGO	Exfoliation at liquid/air interface	Resistive	R.T.	~6%	—	4.3–75.7%	4 s/10 s	—	[149]
GQDs	Hydrothermal	Resistive	24 °C	~390	High (CO, H_2_, CH_4_, CO_2_)	1–100%	12 s/43 s	—	[151]
GQDs	Pyrolysis of citric acid	Resistive	R.T.	—	—	15–80%	~5 s	—	[152]

**Table 4:** Summary of the sensing performance of humidity sensors based on chemical modified graphene.

Material	Preparation Method	Type	Operating Temperature	Sensitivity/Response	Selectivity	Humidity Range	Response/Recovery Time	Stability	Ref.
Graphene/methyl red	Electro-hydrodynamic	Resistive Capacitive	R.T.	96.36% (R) 2,869,500% (C)	—	5–95%	0.251 s/0.35 s	—	[161]
HFBI-rGO	Drop casting	Resistive	R.T.	2.24/RH	Medium (Ethanol, acetone, hexane)	2–50%	130 s/200 s	—	[162]
Pyranine-rGO	Supramolecular assembly	Impedance	25 °C	6000	—	11–95%	<2 s/<6 s	100 cycles	[163]
PEI-GO	Solution casting	Impedance	30 °C	—	—	40–90%	—	—	[156]
Diamine-GO	Brush coating	Impedance	15–35 °C	0.0545 log Z/% RH	—	20–90%	52 s/72 s	54 days	[157]
GO-NH_2_	Drop casting	Conductance	25 °C	870 ± 90	—	5–95%	—	—	[158]
Ag-NA-rGO	Drop casting	Impedance	25 °C	600	—	11–95%	1 s/1 s	110 days	[159]

**Table 5:** Summary of the sensing performance of humidity sensors based on graphene/polymer composites.

Material	Preparation Method	Type	Operating Temperature	Sensitivity/Response	Selectivity	Humidity Range	Response/Recovery Time	Stability	Ref.
m-r(CNC/GO)	Layer-by-layer spraying	Resistive	25 °C	~55	—	20–100%	~10 s/~5 s	—	[166]
CNC/GO	Pour drying	Capacitive	25–45 °C	547	—	25–90%	—	—	[167]
PVA/NFC/rGO	Pour drying	Resistive	R.T.	0.347/%RH	—	30–98%	<2 min	—	[168]
rGO/cellulose	Pour casting	Resistive	23 °C	~40%	—	35–90%	—	—	[169]
rGO/LS	Vacuum filtration	Resistive	25 °C	298%	—	22–97%	100 s/100 s	30 days	[170]
PDA/rGO	Drop casting	Resistive	25 °C	20,000	—	0–97%	20 ms/17 ms	60 days	[176]
PDA/PVA/rGO	Pour drying	Resistive	30 °C	—	—	40–100%	—	—	[175]
PDDA/rGO PSSNa/rGO	Dip coating	Impedance	22–25 °C	1000% (0.2–30%) 300% (0.2–30%)	—	0.2–90%	16 s/24 s 38 s/70 s	—	[171]
QC-P4VP/rGO	Dip-coating	Impedance	20–40 °C	500% (0.18–2.1%)	—	0.18–98%	21 s/78 s	—	[172]
PDDA/rGO	Layer-by-layer self-assembly	Resistive	25 °C	8.69–37.43%	—	11–97%	108–147 s/ 94–133 s	60 days	[173]
PDDA/GO	Layer-by-layer self-assembly	Capacitive	25 °C	1552.3 pF/% RH	—	11–97%	1 s/1 s	60 days	[174]
rGO/PVP	Spin coating	Resistive	R.T.	—	—	30–90%	3 s/3 s	—	[177]
Graphene/PVP	Ink-jet printing	Resistive	R.T.	0.3–0.21%/%RH	—	10–80%	6–16 s/ 60–300 s	28 days	[178]
rGO/PVP	Spary coating	Resistive	20–60 °C	~7	High (HCHO, H_2_S, NH_3_, acetone, H_2_)	7.0–97.3%	2.8s/3.5s	28 days	[179]
PVDF/graphene	Electro-spinning	Capacitive	25 °C	0.0463pF/% RH	—	40–90%	~1000 s/21.3 s	—	[180]
Graphene/PPy	Dip coating	Impedance	R.T.	138	—	12–90%	15 s/20 s	—	[181]
rGO/PU	Spin coating	Resistive	R.T.	—	—	10–70%	3.5 s/7 s	—	[182]
MGO/Nafion	Drop casting	Impedance	R.T.	—	—	11–97%	100–300 s/ 100–300 s	2 months	[183]
GO/C60	Drop casting	Capacitive	R.T.	2770%	—	11–97%	8 s/7 s	21 days	[184]
GO/MWCNT	Drop casting	Capacitive	25 °C	7980 pF/% RH	—	11–97%	5 s/2.5 s	—	[185]

**Table 6:** Summary of the sensing performance of humidity sensors based on graphene/metal, metal oxide, or 2D material composites.

Material	Preparation Method	Type	Operating Temperature	Sensitivity/Response	Selectivity	Humidity Range	Response/Recovery Time	Stability	Ref.
Graphene/SnOx/CF	Electro-spinning	Resistive	20.5 °C	6.22	—	30–80%	8 s/6 s	—	[186]
SnO_2_@G-GO	Electro-spinning	Impedance	20 °C	32 MΩ/% RH	—	30–90%	1 s/1 s	3 cycles	[187]
SnO_2_/rGO	Hydrothermal	Resistive	R.T.	15.19–45.02%	—	11–97%	<100 s/<100 s	60 days	[188]
SnO_2_/rGO	Hydrothermal	Capacitive	25 °C	1604.89 pF/%RH	—	11–97%	6–102 s/6–9 s	—	[77]
rGO/Fe:SnO_2_	Electrostatic interaction	Resistive	R.T.	3.23	—	0–100%	—	—	[189]
CuO/rGO	Hydrothermal	Impedance	25 °C	22,700	—	11–98%	2 s/17 s	—	[190]
Graphene/TiO_2_	Sol–gel	Impedance	25 °C	151	High (NH_3_, NO_2_, NO, CO)	12–90%	128 s/68 s	—	[191]
ZnO/GO	Layer-by-layer self-assembly	Capacitive	R.T.	17785.6 pF/%RH	—	0–97%	~50 s/~20 s	30 days	[192]
Graphene/ZnO	Spin coating	Impedance	R.T.	—	—	0–85%	1 s/2 s	—	[193]
TiO_2_/GO	Drop casting	Resistive	R.T.	> 10^6^	High (H_2_, CO, CH_4_, NO_2_)	9–90%	~70 s	2 months	[194]
rGO–Ag scroll	Molecular combing	Resistive	R.T.	908–1243	—	11–97%	50 s/13 s	30 days	[195]
Au/GO/silica	Sol-gel	Impedance	15–35 °C	−0.0281 log Z/%RH	—	20–90%	119 s/125 s	15 days	[196]
Cu-embeded rGO fiber	Wet spinning	Resistive	20 °C	0.94	—	34–75%	—	—	[197]
rGO/MoS_2_	Mixing and drop casting	Resistive	27 °C	2494.25%	High (H_2_, CH_3_COCH_3_, NO_2_, NH_3_)	5–85%	6.3 s/30.8 s	20 months	[199]
MoS_2_/GO	Mixing and drop casting	Resistive	R.T.	1600	—	25–85%	43 s/37 s	90 days	[198]
rGO/MoS_2_	Hydrothermal	Resistive	R.T.	~25%	High (NO_2_, NH_3_, H_2_, C_2_H_5_OH)	10–90%	30 s/253 s	—	[200]
WS_2_/GO	Mixing and drop casting	Resistive	25 °C	590 0.044/%RH	—	40–80%	25 s/29 s	28 days	[201]
Black P/ graphene	CVD-G electrospray BP	Resistive	R.T.	43.4%	—	15–70%	9 s/30 s	2 weeks	[202]

**Table 7:** Performance of the selected graphene-based humidity sensors with superior characteristics.

Material	Type	Response	Selectivity	Humidity Range	Limit of Detection	Response Time	Recovery Time	Stability	Ref.
GO	Impedance	~10^2 (1)^	—	10–90%	10% ^(2)^	~30 ms	~30 ms	72h	[74]
Ultralarge GO	Conductance	4339 ± 433	—	7–100%	7% ^(2)^	0.2 s	0.7 s	5 days	[121]
rGO/GO/rGO	Voltage	142.5	High (H_2_, hexane, ethanol)	6.3–100%	6.3% ^(2)^	1.9 s	3.9 s	> 1 year	[131]
rGO	Impedance	~10^3 (1)^	—	11–95%	11% ^(2)^	1 s	24 s	30 days	[142]
Graphene/methyl red	Resistive Capacitive	96.36% (R) 2869500% (C)	—	5–95%	5% ^(2)^	0.251 s	0.35 s	—	[161]
PDA/rGO	Resistive	20000	—	0–97%	10% ^(1)^	20 ms	17 ms	60 days	[176]
rGO/MoS_2_	Resistive	2494.25%	High (H_2_, CH_3_COCH_3_, NO_2_, NH_3_)	5–85%	5% ^(2)^	6.3 s	30.8 s	20 months	[199]
PDDA/rGO PSSNa/rGO	Impedance	1000% (0.2–30%) 300% (0.2–30%)	—	0.2–90%	0.2%	16 s 38 s	24 s 70 s	—	[171]
QC-P4VP/rGO	Impedance	500% (0.18–2.1%)	—	0.18–98%	0.18%	21 s	78 s	—	[172]

^(1)^ Estimated from the response curve; ^(2)^ the lowest RH in the sensing measurements.
